# Meta-Analysis of the *INSIG2* Association with Obesity Including 74,345 Individuals: Does Heterogeneity of Estimates Relate to Study Design?

**DOI:** 10.1371/journal.pgen.1000694

**Published:** 2009-10-23

**Authors:** Iris M. Heid, Cornelia Huth, Ruth J. F. Loos, Florian Kronenberg, Vera Adamkova, Sonia S. Anand, Kristin Ardlie, Heike Biebermann, Peter Bjerregaard, Heiner Boeing, Claude Bouchard, Marina Ciullo, Jackie A. Cooper, Dolores Corella, Christian Dina, James C. Engert, Eva Fisher, Francesc Francès, Philippe Froguel, Johannes Hebebrand, Robert A. Hegele, Anke Hinney, Margret R. Hoehe, Frank B. Hu, Jaroslav A. Hubacek, Steve E. Humphries, Steven C. Hunt, Thomas Illig, Marjo-Riita Järvelin, Marika Kaakinen, Barbara Kollerits, Heiko Krude, Jitender Kumar, Leslie A. Lange, Birgit Langer, Shengxu Li, Andreas Luchner, Helen N. Lyon, David Meyre, Karen L. Mohlke, Vincent Mooser, Almut Nebel, Thuy Trang Nguyen, Bernhard Paulweber, Louis Perusse, Lu Qi, Tuomo Rankinen, Dieter Rosskopf, Stefan Schreiber, Shantanu Sengupta, Rossella Sorice, Anita Suk, Gudmar Thorleifsson, Unnur Thorsteinsdottir, Henry Völzke, Karani S. Vimaleswaran, Nicholas J. Wareham, Dawn Waterworth, Salim Yusuf, Cecilia Lindgren, Mark I. McCarthy, Christoph Lange, Joel N. Hirschhorn, Nan Laird, H.-Erich Wichmann

**Affiliations:** 1 Helmholtz Zentrum München, German Research Center for Environmental Health, Neuherberg, Germany; 2 Department of Epidemiology and Preventive Medicine, University Medical Center Regensburg, Regensburg, Germany; 3 Medical Research Council Epidemiology Unit, Institute of Metabolic Science, Cambridge, United Kingdom; 4 Division of Genetic Epidemiology, Department of Medical Genetics, Molecular and Clinical Pharmacology, Innsbruck Medical University, Innsbruck, Austria; 5 Institute for Clinical and Experimental Medicine, Prague, Czech Republic; 6 Department of Medicine and Clinical Epidemiology and Biostatistics, McMaster University, Hamilton, Ontario, Canada; 7 Broad Institute of Harvard and MIT, Cambridge, Massachusetts, United States of America; 8 Charité Campus Virchow Klinikum, Institut für Experimentelle Pädiatrische Endokrinologie, Berlin, Germany; 9 University of Southern Denmark, Copenhagen, Denmark; 10 Department of Epidemiology, German Institute of Human Nutrition Potsdam–Rehbrücke, Nuthetal, Germany; 11 Human Genomics Laboratory, Pennington Biomedical Research Center, Baton Rouge, Louisiana, United States of America; 12 Institute of Genetics and Biophysics ‘A.Buzzati-Traverso’, CNR, Naples, Italy; 13 Centre for Cardiovascular Genetics, Royal Free and University College Medical School, London, United Kingdom; 14 Preventive Medicine Department, University of Valencia, Valencia, Spain; 15 CIBER Fisiopatología de la Obesidad y Nutrición, Instituto de Salud Carlos III, Valencia, Spain; 16 Pasteur Institute, CNRS 8090–Institute of Biology, Lille, France; 17 Institut du Thorax, CNRS ERL3147, INSERM U 915, Nantes, France; 18 Departments of Medicine and Human Genetics, McGill University, Montréal, Québec, Canada; 19 Department of Child and Adolescent Psychiatry, University of Duisburg–Essen, Essen, Germany; 20 Robarts Research Institute, University of Western Ontario, London, Ontario, Canada; 21 Max Planck Institut for Molecular Genetics, Berlin, Germany; 22 Department of Nutrition, Harvard School of Public Health, Boston, Massachusetts, United States of America; 23 Channing Laboratory, Brigham and Women's Hospital and Harvard Medical School, Boston, Massachusetts, United States of America; 24 Cardiovascular Research Center, Prague, Czech Republic; 25 Cardiovascular Genetics Division, University of Utah School of Medicine, Salt Lake City, Utah, United States of America; 26 Department of Epidemiology and Public Health, Imperial College, London, United Kingdom; 27 National Public Health Institute, Department of Child and Adolescent Health, Oulu, Finland; 28 Institute of Health Sciences and Biocenter Oulu, University of Oulu, Oulu, Finland; 29 Institute of Genomics and Integrative Biology, Delhi, India; 30 Department of Genetics, University of North Carolina, Chapel Hill, North Carolina, United States of America; 31 Department of Internal Medicine II, University Medical Center Regensburg, Regensburg, Germany; 32 Children's Hospital, Boston, Massachusetts, United States of America; 33 Genetics Division, Drug Discovery, GlaxoSmithKline, King of Prussia, Pennsylvania, United States of America; 34 Institute of Clinical Molecular Biology, Christian-Albrechts-University of Kiel, Kiel, Germany; 35 Institut fuer Medizinische Biometrie und Epidemiologie, Philipps-Universitaet Marburg, Marburg, Germany; 36 First Department of Internal Medicine, St. Johann Spital, Paracelsus Private Medical University Salzburg, Salzburg, Austria; 37 Faculty of Medicine, Department of Social and Preventive Medicine, Université Laval, Québec, Québec, Canada; 38 Department of Pharmacology, Ernst Moritz Arndt University Greifswald, Greifswald, Germany; 39 deCODE genetics, Reykjavik, Iceland; 40 Faculty of Medicine, University of Iceland, Reykjavik, Iceland; 41 Department of Community Medicine, Ernst Moritz Arndt University Greifswald, Greifswald, Germany; 42 Welcome Trust Center for Human Genetics, University of Oxford, Oxford, United Kingdom; 43 Oxford Center of Diabetes, Endocrinology and Metabolism, The Churchill Hospital, Headington, Oxford, United Kingdom; 44 Department of Biostatistics, Harvard School of Public Health, Boston, Massachusetts, United States of America; 45 Chair of Epidemiology, Institute of Medical Informatics, Biometry and Epidemiology, Ludwig-Maximilians-Universität, Munich, Germany; 46 Klinikum Grosshadern, Munich, Germany; University of Alabama at Birmingham, United States of America

## Abstract

The *INSIG2* rs7566605 polymorphism was identified for obesity (BMI≥30 kg/m^2^) in one of the first genome-wide association studies, but replications were inconsistent. We collected statistics from 34 studies (n = 74,345), including general population (GP) studies, population-based studies with subjects selected for conditions related to a better health status (‘healthy population’, HP), and obesity studies (OB). We tested five hypotheses to explore potential sources of heterogeneity. The meta-analysis of 27 studies on Caucasian adults (n = 66,213) combining the different study designs did not support overall association of the CC-genotype with obesity, yielding an odds ratio (OR) of 1.05 (p-value = 0.27). The I^2^ measure of 41% (p-value = 0.015) indicated between-study heterogeneity. Restricting to GP studies resulted in a declined I^2^ measure of 11% (p-value = 0.33) and an OR of 1.10 (p-value = 0.015). Regarding the five hypotheses, our data showed (a) some difference between GP and HP studies (p-value = 0.012) and (b) an association in extreme comparisons (BMI≥32.5, 35.0, 37.5, 40.0 kg/m^2^ versus BMI<25 kg/m^2^) yielding ORs of 1.16, 1.18, 1.22, or 1.27 (p-values 0.001 to 0.003), which was also underscored by significantly increased CC-genotype frequencies across BMI categories (10.4% to 12.5%, p-value for trend = 0.0002). We did not find evidence for differential ORs (c) among studies with higher than average obesity prevalence compared to lower, (d) among studies with BMI assessment after the year 2000 compared to those before, or (e) among studies from older populations compared to younger. Analysis of non-Caucasian adults (n = 4889) or children (n = 3243) yielded ORs of 1.01 (p-value = 0.94) or 1.15 (p-value = 0.22), respectively. There was no evidence for overall association of the rs7566605 polymorphism with obesity. Our data suggested an association with extreme degrees of obesity, and consequently heterogeneous effects from different study designs may mask an underlying association when unaccounted for. The importance of study design might be under-recognized in gene discovery and association replication so far.

## Author Summary

A polymorphism of the *INSIG2* gene was identified as being associated with obesity in one of the first genome-wide association studies. However, this association has since then been highly debated upon inconsistent subsequent reports. We collected association information from 34 studies including a total of 74,000 participants. In a meta-analysis of the 27 studies including 66,000 Caucasian adults, we found no overall association of this polymorphism rs7566605 with obesity, comparing subjects with a body-mass-index (BMI)≥30 kg/m^2^ with normal BMI subjects (BMI<30 kg/m^2^). Our data suggested an association of this polymorphism with extreme obesity (e.g., BMI≥37.5 kg/m^2^) compared to normal controls. Such an association with extreme obesity might induce heterogeneous effects from different study designs depending on the proportion of extreme obesity included by the design. However, further studies would be required to substantiate this finding. The importance of study design might be under-recognized in gene discovery and association replication so far.

## Introduction

One of the first genome-wide association (GWA) studies ever and the first on obesity identified the *INSIG2* gene represented by the rs7566605 polymorphism as a novel gene for common obesity [1]. Functional evidence depicted the *INSIG2* gene from the very start as an interesting candidate for obesity as being involved in the reversed cholesterol transport by an interaction with sterol regulatory element-binding proteins (SREBPs) [2], which are transcription factors that activate the synthesis of cholesterol and fatty acids in the liver and other organs [3].

The observed SNP-obesity-association was replicated in some, but not in all studies [4]–[11]. A letter to *Science* by the authors of the initial report in response to the emerging debate of the early inconsistent results [1] raised the question of whether the association might be more pronounced in studies that were not ascertained for reasons related to better health status, when comparing more severely obese subjects with normal controls, in populations with a higher prevalence of obesity or in populations with a higher mean age. The need for a meta-analysis was stated there for the first time and re-stated by Lyon and colleagues [12]. Furthermore, a secular trend for increasing prevalence of obesity was observed in two large population-based studies from the same geographical region using the same protocols but one recruited 1994/95 (KORA-S3) and the other 1999–2001 (KORA-S4) [13]. The later study showed a stronger *INSIG2*-obesity-association compared to the earlier study: This raised the additional question whether the changes in nutritional intake and physical activity [14],[15] believed to contribute to the increase in the prevalence of obesity during the last decades were the reason for some of the between-study heterogeneity observed for this SNP's association with obesity.

The inconsistent reported associations and the many resulting debates motivated us to undertake a systematic meta-analysis of all available data to investigate potential causes of heterogeneity and to look for consistent results among subgroups. It was thus the specific aim of this meta-analysis to explore five hypotheses for heterogeneity of the rs7566605 association with obesity: (*Hypothesis 1*) The association depends on study design. Therefore, we classified studies as general population-based (GP) when they were neither selected for any disease nor for not having any disease, as any selection of this type was shown to potentially induce bias for outcomes associated with the disease [16]. We classified studies as ‘healthy population’ (HP) when they were selected for reasons related to a better health status (i.e. studies including subjects from working populations or studies excluding subjects for with diseases such as diagnosed type 2 diabetes), or obesity studies (OB) when they were specifically designed to investigate obesity, usually case-control or family studies. We did not include studies that were ascertained for any disease to reduce overly complexity. (*Hypothesis 2*) The association is more pronounced when comparing more extreme cases of obesity with normal or lean subjects, or (*Hypothesis 3*) among studies with a greater percentage of obese individuals. (*Hypothesis* 4) The association is differentially seen in studies including subjects with a higher age compared to studies based on younger populations, or (*Hypothesis 5*) more pronounced in studies with a more recent assessment of BMI (after year 2000) assuming that these studies would reflect the changes in dietary habits and physical activity of the last decade and assuming that subjects with the *INSIG2* risk genotype are more prone to gain weight in such an environment.

## Results

### Data Collected

We have gathered data on 34 studies from across Europe, North America, and Asia that met the inclusion criteria including a total of 74,345 subjects. All studies were categorized *a priori* according to their study design (GP, HP, OB), ethnicity, and whether it was an adult or children population (Tables S1 and S2). A more detailed description can be found in the original study publications [1], .

### Main Analysis

The main results are summarized in Table 1: The meta-analysis of 27 studies of Caucasian adults (n = 66,213) showed a fixed effect odds ratio (OR) of 1.076 (p-value = 0.023) and a random effects OR of 1.051 (p-value = 0.268) with the I^2^ measure indicating significant between-study heterogeneity (I^2^ = 41.0%, 95% confidence interval, CI = [6.6%, 62.8%], Q-test p-value = 0.015).

**Table 1:** Main meta-analysis results of the *INSIG2* rs7566605 association with obesity.

	# cases/controls (# studies)	OR (p-value) fixed effect	OR (p-value) random effect	I^2^ (p-value)	P-value test for difference of fixed [random] effect ORs^b^
All-CA	16,365/49,848 (27)	1.076 (0.023)	1.051 (0.268)	41.0 (0.015)	
All-CA^a^	10,761/30,168 (19)	1.077 (0.074)	1.054 (0.361)	36.4 (0.057)	
GP	9162/39,682 (16)	1.097 (0.015)	1.092 (0.035)	10.9 (0.329)	GP vs HP: 0.004 [0.005]
GP^a^	5803/23,243 (12)	1.100 (0.054)	1.080 (0.060)	1.3 (0.431)	GP^a^ vs HP^a^: 0.038 [0.089]
HP	1307/6333 (5)	0.796 (0.028)	0.796 (0.028)	0.0 (0.415)	HP vs OB: 0.038 [0.022]
HP^a^	795/4116 (3)	0.821 (0.135)	0.772 (0.198)	46.2 (0.156)	HP^a^ vs OB^a^:0.038 [0.109]
OB	5896/3833 (6)	1.163 (0.018)	1.152 (0.253)	63.2 (0.018)	OB vs GP: 0.478 [0.680]
OB^a^	3119/2338 (4)	1.155 (0.144)	1.179 (0.337)	65.0 (0.036)	OB^a^ vs GP^a^: 0.657 [0.693]
ALL-NC	553/4336 (4)	1.013 (0.936)	0.945 (0.818)	43.5 (0.150)	
ALL-CH	1802/1441 (3)	1.147 (0.216)	1.147 (0.216)	0.0 (0.830)	

Pooled association for all Caucasian adult studies combined (All-CA) as well as stratified by study type (GP = general population, HP = healthy population, OB = obesity study), for all non-Caucasian studies (All-NC), or the children studies (All-CH) indicating differential results among GP or HP studies (*Hypothesis 1*). Numbers stated are the number of obese (BMI≥30 kg/m^2^, ‘cases’) and non-obese (BMI<30 kg/m^2^, ‘controls’) subjects with the number of studies, the recessive model ORs (p-value) comparing the odds for obesity among the subjects with the CC genotype versus subjects with the CG or GG genotype using fixed or random effects models, the I^2^ measure (p-value of the Q-statistics), and the p-value testing for pair-wise difference between GP, HP, and OB studies.

a Excluding studies published before the response letter in *Science* by Herbert et al., December 2006 [1], in which the hypothesis of potential heterogeneity due to study design and a first call for this meta-analysis were stated (i.e., excluding American_Polish, NHS, KORA_S4, Essen_trios, EPIC_Norfolk, MRC_Ely, DESIR, SHIP, OB_adult).

b P-values corrected for pair-wise comparison of three subgroups need to be multiplied by three.

OR = Odds Ratios.

### Some Heterogeneity Might Relate to Study Type

The I^2^ measure declined (I^2^ = 10.9%, 95% CI = [0.0%, 48.1%], Q-test p-value = 0.329) by restricting to the 16 GP studies, including 48,844 subjects, yielding a fixed [random] effects OR of 1.097 [1.092] (p-value = 0.015 [0.035]). The OR estimates were similar when excluding early published studies (p = 0.054 [0.060]).

The I^2^ measure for heterogeneity was zero among the five HP studies, including 7640 subjects, and combined estimates yielded a tendency towards a protective OR of 0.796 (p-value = 0.028) without remarkable change when excluding the early published studies.

The OR among the six OB studies, including 9729 subjects, of 1.152 using the random effect model was higher than the one for the GP studies, but not statistically significantly different from unity (p-value = 0.253). There was substantial heterogeneity among the OB studies (I^2^ = 63.2%, Q-test p-value = 0.018). The OR estimates were similar when excluding the early published studies.

**Figure 1:**
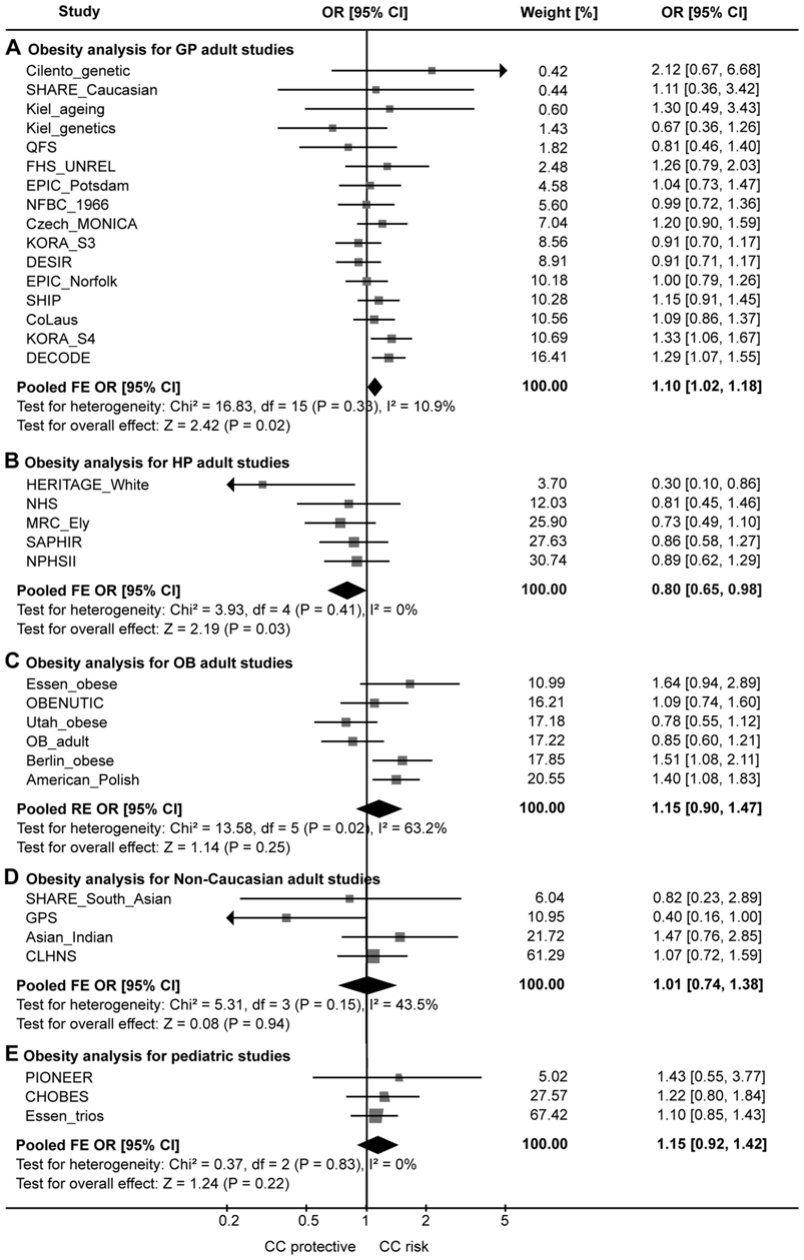
Forest plot on Odds Ratio estimates of the *INSIG2* rs7566605 association with obesity. Association with obesity (BMI ≥ 30 kg/m^2^) for (A) the Caucasian adult general population-based (GP) studies, (B) the Caucasian adult studies ascertained for reasons related to health status (HP), (C) the Caucasian adult obesity case-control studies (OB), (D) the non-Caucasian adult studies, and (E) the pediatric studies. Shown are recessive model OR estimates comparing the CC genotype versus CG or GG for each study and pooled estimates. The fixed effect (FE) model OR is shown in case of no significant heterogeneity as tested by the Q-statistics; a random effect (RE) model is shown otherwise.

Figure 1A–1C shows forest plots of the Caucasian adult studies with combined estimates by study type. The p-value testing for difference between the GP and the HP combined estimates was 0.004 [0.012 when corrected for pair-wise testing of three subgroups] and 0.039 [0.089] when excluding the early published studies. Thus, there is some, but not completely conclusive evidence for *Hypothesis 1* that study design might explain some of the between-study heterogeneity of this genetic association.

### Studies in Non-Caucasian Adults or Children

In the pooled analysis of the four studies on non-Caucasian adults (n = 4889), we found no significant association of the CC genotype with obesity. The pooled analysis of the three pediatric studies (n = 3243) was also not significant (Table 1, Figure 1D and 1E).

### Increasing Strength of Association in More Extreme Comparisons

Our data suggested an association with increased ORs among the Caucasian adults when more extremely obese subjects were compared to lean controls (Table S3; *Hypothesis 2*): combining over all studies, the ORs gradually increased from 1.156 to 1.183, 1.221, or 1.265 (p-values ranging from 0.001 to 0.003) when moving the BMI cut-off for the obese cases from 32.5 to 35.0, 37.5 or 40.0 kg/m^2^, respectively, and comparing to controls with BMI<25 kg/m^2^. A similar trend for increasing ORs was seen when comparing extremely obese subjects against controls with BMI<30 kg/m^2^ or BMI<20.0 kg/m^2^. Among the GP studies, the ORs increased from 1.198 to 1.257, 1.313, or 1.414 (p-values ranging from 0.001 to 0.003), respectively. The analogous comparison for HP studies revealed that the protective influence of the CC homozygous was reversed in the more extreme comparisons with ORs from 0.856 to 0.959, 1.139, or 1.604. For the OB studies, the ORs of the analogous comparisons were well above unity for all comparisons, but did not show a trend.

As summarized in Table 2, the accompanying trend analyses of the CC genotype frequencies across the various BMI categories indicated significantly increased CC genotype frequencies from 10.4% to 12.5% (p-value testing for trend = 0.0002), which persisted when excluding the early published studies (p-value = 0.0008). A similar trend was seen among GP studies.

**Table 2:** Comparing more extreme degrees of obesity.

BMI (kg/m^2^)			<20	20–25	25–30	30–32.5	32.5–35	35–37.5	37.5–40	>40	P-value^b^
All-CA	%CC		10.4	10.7	10.5	10.5	11.9	11.2	12.0	12.5	0.0002
	N	66,213	3314	22462	23072	5869	3578	2150	1404	3364	
All-CA^a^	%CC		9.9	10.8	10.9	10.8	11.6	12.0	12.1	12.7	0.0008
	N	39,414	2321	13671	3447	1962	1962	1162	829	2317	
GP	%CC		10.7	10.8	10.2	10.4	12.0	12.4	12.6	13.5	0.007
	N	48,844	2490	18551	18641	4300	2299	1111	620	832	
GP^a^	%CC		10.6	10.8	10.8	10.7	12.01	12.9	12.1	13.7	0.005
	N	29,046	1684	10975	10584	2546	1432	727	421	677	
HP	%CC		10.6	10.4	11.9	9.7	8.1	7.5	13.0	8.6	0.345
	N	7640	273	2772	3288	679	323	147	77	81	
HP^a^	%CC		8.3	10.1	11.8	10.1	7.0	9.6	17.6	2.9	0.758
	N	4911	121	1716	2279	457	186	83	34	35	
OB	%CC		8.5	10.3	10.8	11.8	13.1	10.3	11.3	12.4	0.009
	N	9729	551	2139	1143	890	956	892	707	2451	
OB^a^	%CC		8.1	11.9	10.0	12.4	12.2	10.8	11.5	12.4	0.054
	N	5457	516	980	842	444	344	352	374	1605	

The data suggest an association when comparing more extreme degrees of obesity with normal controls, which is a potential explanation for the heterogeneity of the *INSIG2* rs7566605 association with obesity (*Hypothesis 2*). Numbers stated are frequencies of risk genotype CC (C being the minor allele) across BMI categories for the Caucasian adult studies combined (All-CA) as well as stratified by study type (GP = general population, HP = healthy population, OB = obesity study). Also given are the p-values from testing for a trend of genotype frequencies across categories. (Not for All-NC or All-CH due to the few subjects in each category.)

a Excluding studies published before the response letter by Herbert et al., December 2006 [1] in which the hypothesis of potential heterogeneity due to study design and a first call for this meta-analysis were stated (i.e., excluding American_Polish, NHS, KORA_S4, Essen_trios, EPIC_Norfolk, MRC_Ely, DESIR, SHIP, OB_adult, which are all Caucasian studies).

b P-value testing for a trend across the BMI categories.

In both types of analyses, the varying cut-point ORs as well as the trend in genotype frequencies by BMI categories, suggest an association of the rs7566605 with extreme obesity compared to normal controls (*Hypothesis 2*).

### Further Hypotheses on Potential Sources of Heterogeneity

Table 3 summarizes the results of the further three hypotheses to explain heterogeneity, which were tested for the Caucasian adult studies. *Hypothesis 3*: there was some tendency towards higher ORs among ‘more obese’ study populations compared to the ‘less obese’ study populations (p-value = 0.052 [0.285] testing for difference of the fixed [random] effects ORs), but not statistically significant. *Hypothesis 4*: there was no evidence for any difference between studies from older populations (i.e. mean age of subjects above 50 years) as compared to studies from younger populations (i.e. mean age below 50 years). *Hypothesis 5*: there was a tendency towards more pronounced ORs for the studies with BMI assessed after the year 2000 as compared to studies with BMI assessed before 2000 (p-value = 0.007 [0.095]), but not statistically significant given the various tests performed and particularly not when excluding the early published studies (p-value = 0.086 [0.248]). *Hypotheses 3–5* were not tested in the HP or OB stratified analyses as too few (3–6) studies were available.

**Table 3:** Exploring potential sources of heterogeneity for the *INSIG2* rs7566605 association with obesity (*Hypotheses 3–5*).

Group		# subjects (# studies)	OR (p-value) fixed effect	OR (p-value) random effect	I^2^ (p-value)	Testing for difference p-value^e^
% obese ≥18%^a^	All-CA	34,999 (17)	1.127 (0.003)	1.083 (0.223)	55.0 (0.003)	
	All-CA^d^	20,882 (12)	1.108 (0.049)	1.069 (0.444)	54.0 (0.013)	
	GP	21,394 (8)	1.175 (0.002)	1.164 (0.013)	21.2 (0.261)	
	GP^d^	13,232 (6)	1.139 (0.044)	1.113 (0.251)	33.3 (0.186)	
% obese <18%^a^	All-CA	31,214 (10)	0.989 (0.841)	0.989 (0.841)	0.0 (0.856)	0.052 [0.285]
	All-CA^d^	18,532 (7)	1.024 (0.737)	1.024 (0.737)	0.0 (0.731)	0.368 [0.699]
	GP	27,450 (8)	1.006 (0.912)	1.006 (0.912)	0.0 (0.792)	0.044 [0.083]
	GP^d^	15,814 (6)	1.048 (0.539)	1.048 (0.539)	0.0 (0.709)	0.401 [0.615]
Mean age ≥50 years^b^	All-CA	28,807 (11)	1.098 (0.059)	1.056 (0.464)	41.9 (0.070)	
	All-CA^d^	17,635 (8)	1.110 (0.094)	1.068 (0.432)	25.1 (0.228)	
	GP	19,953 (7)	1.131 (0.041)	1.120 (0.114)	14.1 (0.322)	
	GP^d^	13,221 (6)	1.181 (0.017)	1.169 (0.054)	9.0 (0.358)	
Mean age <50 years^b^	All-CA	37,406 (16)	1.060 (0.166)	1.047 (0.442)	43.6 (0.032)	0.593 [0.929]
	All-CA^d^	21,779 (11)	1.051 (0.375)	1.050 (0.549)	46.2 (0.046)	0.521 [0.881]
	GP	28,891 (9)	1.074 (0.146)	1.071 (0.204)	14.9 (0.310)	0.505 [0.621]
	GP^d^	15,825 (6)	1.025 (0.728)	1.025 (0.728)	0.0 (0.613)	0.149 [0.217]
BMI after/during 2000^c^	All-CA	28,810 (14)	1.172 (0.0004)	1.135 (0.071)	47.5 (0.025)	
	All-CA^d^	20,374 (11)	1.145 (0.014)	1.119 (0.164)	38.9 (0.089)	
	GP	17,217 (7)	1.218 (0.001)	1.216 (0.002)	3.9 (0.396)	
	GP^d^	13,221 (6)	1.181 (0.017)	1.169 (0.054)	9.0 (0.358)	
BMI before year 2000^c^	All-CA	37,403 (13)	0.987 (0.771)	0.986 (0.767)	1.5 (0.431)	0.007 [0.095]
	All-CA^d^	19,040 (8)	0.990 (0.875)	0.984 (0.836)	22.5 (0.250)	0.086 [0.248]
	GP	27,450 (8)	1.021 (0.676)	1.021 (0.676)	0.0 (0.713)	0.023 [0.029]
	GP^d^	15,825 (6)	1.025 (0.728)	1.025 (0.728)	0.0 (0.613)	0.149 [0.217]

Stated values are ORs (p-values) based on fixed or random effects models and measures of I^2^ (p-value from Q statistics) for each group and p-values testing for difference between ORs of the two corresponding groups. (Not for HP, OB, NC, or CH studies due to the few number of studies.)

a Studies with percentage of subjects with BMI ≥30≥18% or <18%.

b Studies with mean age ≥50 years or <50 years.

c Studies with BMI assessment after/during year 2000 or before year 2000.

d Excluding studies published before the response letter by Herbert et al., December 2006 [1], in which the hypothesis of potential heterogeneity due to study design and a first call for a meta-analysis were stated (i.e. excluding American_Polish, NHS, KORA-S4, Essen_trios, EPIC_Norfolk, MRC_Ely, DESIR, SHIP, OB_adult).

e Testing for difference of fixed effect OR [random effects OR].

OR = Odds Ratio, All-CA = all Caucasian adult studies combined, GP = general population studies.

### Sensitivity Analyses

The sensitivity analyses (Table S4) indicated robustness of estimates towards selection of gender or age and no significant difference between published and unpublished studies. Excluding the two studies with self-reported BMI from the overall GP meta-analysis resulted in a slight increase of the OR estimate.

### Genetic Model

We specifically examined the association under the recessive genetic model as suggested by the original paper [1]. Our data on the raw numbers of obese or non-obese subjects with one of the three genotypes underscored a recessive model in the Caucasian adult studies combined (OR_CCversusGG_ = 1.112 [1.029, 1.203], p-value = 0.007, and OR_GCversusGG_ = 0.988 [0.940, 1.037], p-value = 0.618) as well as among the GP studies (OR_CCversusGG_ = 1.076 [0.976, 1.185], p-value = 0.142, and OR_GCversusGG_ = 0.995 [0.956, 1.036], p-value = 0.813).

### Secondary Analyses on BMI as a Quantitative Outcome

The secondary analyses on BMI as a quantitative outcome were only performed in GP and HP studies. These analyses generally showed results consistent with the obesity analyses, but less, if any, significance (Figure S1, Tables S5, S6, and S7).

## Discussion

We conducted a systematic meta-analysis on published and unpublished studies by collecting summary statistics on the association of the rs7566605 SNP near the *INSIG2* gene using a recessive genetic model as proposed by Herbert and colleagues. This SNP was highly debated and the inconsistent findings were very much puzzling underscored by again inconclusive findings in two recent publications [21],[22]. To solve this puzzle, we collected aggregated study-specific data from 34 studies with a total of 74,345 subjects analyzed according to a standardized model.

The main analysis did not support evidence for an overall association with obesity of the CC-genotype compared to the CG/GG (OR = 1.05, p-value = 0.268). Our data suggested an association for more extreme obese subjects (BMI≥32.5, 35.0, 37.5, 40.0 kg/m^2^) compared to normal controls (BMI<25 kg/m^2^) with ORs increasing to 1.16, 1.18, 1.22, 1.27 (p-values between 0.001 and 0.003) and significantly increased CC-genotype frequencies with increasingly high BMI categories (10.4% to 12.5%, p-value for trend = 0.0002).

The main analysis pointed towards significant between-study heterogeneity with an I^2^ measure of 41%. When we restricted the analysis to GP studies, the I^2^ declined to 11% and the OR increased to 1.10. This is in-line with a very recently published study, which found the OR to increase from 1.02 for a combined analysis of diverse types of studies including 16,781 subjects to an OR of 1.15 when restricting to the general population-based INTER99 cohort including 6,158 subjects [21]. This was the largest GP study on this SNP-association prior to this meta-analysis.

### The Degree of Obesity and the Study Design as a Potential Source of Heterogeneity

The results of our analyses suggest an association of this SNP with extremely obese subjects compared to normal controls, but future research will need to confirm this finding. Study design can impact how many extremely obese subjects are included in the study. Study designs that sample more extremely obese subjects will have greater power to detect the association, while study designs that sample fewer of these subjects will have little power to detect the association. An association with extreme obesity might well be masked by study design, and meta-analyses which disregard study design differences.

The tendency of higher OR estimates observed in the general population-based studies (GP) and the obesity case-control studies compared to ‘healthy population’ (HP) studies could possibly reflect the association for extreme obesity compared to normal controls. We have classified studies as ‘ascertained for criteria related to a better health status’ (‘HP’) when patient groups were excluded or when the sample was ascertained based on working populations, which are known to be usually more healthy. We have performed this classification blinded for the study estimate to exclude bias from informative misclassification. It could be that the common rs7566605 directly or via tagging another possibly rare and quite penetrant variant does not so much alter BMI throughout the distribution, but really puts participants into the very obese category. Thus an effect is picked up in the GP samples, but not in the HP studies with fewer extremely obese persons. This would also be in-line with (i) our more pronounced findings in the studies with a higher percentage of obese subjects, and (ii) the lack of association in the quantitative BMI-analysis, which tests for a shift in the full BMI distribution.

It could also be hypothesized that the between-study heterogeneity is due to an interaction of the gene with the environment of high fat diet: *INSIG2* is regulated by atherogenic diet and oxidized oil in rodents [23],[24] and such a diet relates to higher obesity status. A gene-environment interaction was also suggested by reports that life-style interventions including physical training have less positive effects in CC genotype carriers than in CG/GG subjects [25]–[27]. A person at the brink of getting obese might either comply to exercise and avoid becoming obese or might give up and end in the extreme obesity category. This would be in-line with our pronounced findings for more extreme degrees of obesity. It might also be speculated that our more pronounced association among studies with BMI assessment after the year 2000 compared to before 2000 reflects this gene-environment interaction as well: assuming that a change in nutritional habits and physical activity contributed to the increase in obesity observed in the last decades, the studies with a more recent BMI assessment might reflect this more “modern” environment and the *INSIG2*-obesity association would emerge here more clearly.

Also, unknown epistatic interaction of the rs7566605 with one or other (rare) polymorphisms could lead to association with the more extreme obesity phenotype, with the *INSIG2* gene being part of a complex that functions as a biological entity (*SREBP*, *SCAP*, *INSIG2*).

The importance of ascertainment of the study sample might be under-recognized so far. Monsees and colleagues [16] have illustrated that ascertaining for or against disease would induce a bias in genetic association estimates when the genetic marker as well as the phenotype under study (here obesity) are associated with the disease. As obesity is associated with many chronic diseases such as type 2 diabetes and cardiovascular disease, exclusion of such study participants opens up for bias, if association of the SNP with the disease cannot be precluded. We had specifically excluded studies ascertained for disease and had also planned on separating HP from GP studies ahead of the analyses. We would like to highlight that we have adopted a very strict definition of GP and that there might be special advantages in using either disease-ascertained studies [28] or particularly healthy samples in other instances [29].

### Strengths and Limitations of This Study

This meta-analysis has several strengths: (1) We have conducted a systematic approach by collecting all studies published before January 1, 2008, including seven studies that were unpublished at that time. (2) The meta-analysis is large including a total of 74,365 subjects. (3) We separated working tasks, with one researcher designing the analysis plan, recruiting studies, classifying studies by study type, and deciding upon compliance to inclusion criteria, while the other cared for the incoming data and performed the analysis. Therefore, design decisions were made in a blinded way, which guarded against subtle post-hoc data-driven analysis decisions, study selection bias, and informative misclassification of study design. (4) We collected data according to a strict protocol including standardized analysis from each study partner, with strong quality control of study-specific results. (5) We performed only a limited number of pre-defined subgroup analyses with some amendment during the review process. (6) We had a strong focus on the diversity of study design, which is unique in genetic epidemiological research at the time being and an issue probably under-recognized so far.

It might be considered a disadvantage that we did not include studies with subjects selected for diseases, particularly those associated with type 2 diabetes and thus a higher prevalence of obesity, as the association might be stronger in such studies. This might have been one reason for the initial investigation by Herbert and colleagues to detect this association, as mostly type 2 diabetes or asthma ascertained samples had been used. However, we excluded these samples by design in order to reduce heterogeneity and to reduce the influence of counter-regulating disease processes or medications. Furthermore, publication bias is always a threat for meta-analyses as the extent and direction of this selection cannot fully be determined; we attempted to guard against this by recruiting also unpublished studies. It might be considered a further disadvantage that our hypotheses were motivated by the early published studies, which are included in this meta-analysis; to accommodate for this fact, we repeated all analyses excluding these studies (see Text S1D). Finally, it might be considered a disadvantage that we were not able to recruit enough non-Caucasian adult or children studies for a conclusive comparison with the Caucasian adult studies.

### Conclusions

This pooled analysis including all study designs does not provide evidence for overall association of the *INSIG2* rs7566605 CC genotype with increased risk of obesity compared to the CG or GG genotypes. Our data suggest an association with extreme degrees of obesity and consequently heterogeneous effects from different study designs may mask an underlying association when unaccounted for. The importance of study design might be under-recognized in gene discovery and association replication so far.

## Materials and Methods

### Meta-Analysis Concept

We designed our meta-analysis as a pooled analysis of study-specific association estimates according to a standardized protocol (see ‘data form’, Text S1B, and pre-defined analysis plan, Text S1C) with an amendment added during the review process (Text S1D).

Our eligibility criteria for studies were (i) data available on BMI, the *INSIG2* rs7566605 SNP genotypes, age and sex, (ii) sample size of at least 200 subjects, (iii) ethical approval, and (iv) either general population-based (GP), ascertained for reasons related to a better health status such as studies including only subjects in the work-force or studies excluding subjects with diseases (‘healthy population’, HP), or designed specifically to study obesity such as obesity case-control or obesity family studies (OB). We excluded all studies selecting subjects for any disease. For more information on the classification of studies by study type, see Text S1E. We did not exclude on any age or ethnicity criteria to allow exploration of potential heterogeneity. We controlled for study selection bias by separating the two main tasks between the two first authors: IMH. took care of study recruitment, compliance to inclusion criteria, and classification of studies by study type, and CH performed quality control and statistical analysis.

### Study Recruitment, Collection of Aggregated Data, and Quality Control

We identified all eligible studies published before January 1, 2008 by a systematic PubMed literature search using the search terms ‘INSIG2’ OR ‘INSIG-2’ OR ‘rs7566605’. Additionally we identified unpublished studies through contacting researchers in the field by making a call for this meta-analysis in several consortia (GIANT, KORA-500K, *IL-6*-consortium), in the letter to *Science* by Herbert and colleagues [1], in the paper by Lyon and colleagues [12], and in meeting presentations. We sent out and collected standardized data forms, and verified all entries for within-plausibility as well as consistency with publications, if available. We made plausibility checks by use of double information in the aggregated data. All study-specific ambiguities were clarified with the respective study investigators.

All involved studies were conducted according to the principles expressed in the Declaration of Helsinki. The studies were approved by the local Review Boards. All study participants provided written informed consent for the collection of samples and subsequent analysis.

### Statistical Analysis

For each study, OR estimates comparing the odds of obesity (BMI≥30 kg/m^2^) for subjects with the minor-allele homozygous genotype (CC) with subjects of the other genotypes combined (CG, GG), thus assuming a recessive model, were calculated using logistic regression adjusting for age and sex. We also collected OR estimates with standard errors (SE) for the odds of more extreme degrees of obesity (i.e. subjects with BMI≥32.5, 35.0, 37.5, or 40.0 kg/m^2^) compared to various degrees of leanness (i.e. subjects with BMI<30, 25, or 20 kg/m^2^). Furthermore, we collected summary statistics (mean and SE) on the difference in mean BMI between subjects with the CC genotype compared to subjects with the CG or GG genotypes using linear regression adjusted for age and sex. For each study, analyses stratified for sex or age (with a cut-off at 50 years) were performed as well. Among the six studies from non-Caucasian populations, two studies had too few (<3) subjects among the obese with the CC genotype to be included into the dichotomous obesity analysis, while they were included for the quantitative BMI analysis.

For the meta-analysis, we combined beta-estimates among Caucasian adult studies (All-CA) followed by a stratified analysis by study type (GP, HP, OB) and combined estimates among non-Caucasian (All-NC) or children studies (All-CH), see ‘amendment to analysis plan’, Text S1D.

The following was only performed on Caucasian adults as the number of available non-Caucasians or children was too low. We tested for differential association between the GP, HP, or OB studies applying a t-test on the combined beta-estimates and correcting p-values for testing three subgroups. We tested for a trend in CC genotype frequencies across the different BMI categories and tested for differential associations separating the studies for higher or lower obesity prevalence, higher or lower mean age of study subjects, or for a more or less recent BMI assessment using a t-test on the combined beta-estimates. This was complemented by sensitivity analyses stratifying on sex, age, publication status, and type of BMI assessment. As the hypotheses were motivated by the early published studies mentioned in the letter to *Science* by Herbert and colleagues [1], we repeated all analyses with exclusion of these hypotheses-generating studies.

In all analyses, between-study heterogeneity was tested by the χ^2^-based Q-statistic and quantified by I^2^ as a measure of the proportion of variance between the study-specific estimates that is attributable to between-study difference rather than random variation. We pooled study-specific estimates according to the inverse-variance weighted fixed effect or the DerSimonian and Laird random effects model [30]. Heterogeneity was considered to be significant at the 10% level. All statistical analyses were performed with SAS (statistical analysis software, SAS institute, Inc.). Forest plots were prepared using Review Manager software (Cochrane Collaboration, Copenhagen, DK).

## Supporting Information

Figure S1 Forest plot on estimates of the *INSIG2* rs7566605 association with BMI. Association of the *INSIG2* SNP with BMI for (A) the Caucasian adult general-population based studies (GP), (B) the Caucasian adult studies selected for healthy population (HP), (C) the Non-Caucasian adult studies, (D) the pediatric studies. Shown are recessive model beta estimates comparing the CC genotype versus CG or GG for each study and pooled estimates. The fixed effect (FE) model beta is shown in case of no significant heterogeneity as tested by the Q-statistics; a random effect (RE) model is shown otherwise.
(0.44 MB TIF)

Table S1 Characterization of eligible and recruited studies.
(0.12 MB DOC)

Table S2 More details on study characteristics.
(0.20 MB DOC)

Table S3 Stronger genetic effects when comparing more extreme degrees of obesity as an explanation of the heterogeneity of the *INSIG2* rs7566605 association (*Hypothesis 2*). Pooled association estimates for increasing degrees of obesity for all Caucasian adult studies combined (All-CA) as well as stratified by study type (GP = general population, HP = healthy population, OB = obesity study), for all Non-Caucasian studies (All-NC), but not for children studies due to non-comparability of BMI categories. Numbers stated are ORs comparing cases versus controls (p-values) from the pooled analysis (fixed and random effects), number of cases/controls (number of pooled studies), and the I^2^ (p-value from Q statistics).
(0.07 MB DOC)

Table S4 Sensitivity analyses for the association of the *INSIG2 SNP* with obesity regarding sex, age, published or unpublished status of studies, self-reported or measured BMI. Stated values are ORs (p-values) based on fixed or random effects models, the I^2^ (p-value of Q test) for each group, and p-values testing for difference between the fixed effect [random effect] ORs of the two corresponding groups. (Not for HP, OB, All-NC, or All-CH due to low numbers of studies.)
(0.07 MB DOC)

Table S5 Main results of pooled association of the *INSIG2* SNP with body-mass-index (BMI). The analyses for all Caucasian adult studies combined (All-CA) as well as stratified by study type (GP = general population, HP = healthy population, OB = obesity study), for all Non-Caucasian studies (All-NC), and for the children studies (All-CH) indicated some difference between GP and HP studies (*Hypothesis 1*). Numbers stated are recessive model beta-estimates (p-values), i.e., mean difference of BMI between subjects with the CC genotype compared to subjects with the CG or GG genotype, using fixed or random effects models, the I^2^ (p-value of Q-statistics), and p-values testing for pair-wise difference between GP, HP, or OB studies.
(0.04 MB DOC)

Table S6 Exploring potential sources of heterogeneity of the *INSIG2* rs7566605 association with BMI (*Hypotheses 3–5*). Stated values are pooled beta-estimates (p-values) based on fixed or random effects model, I^2^ (p-values of Q-test) for each group, and p-values testing for difference between the beta-estimates of the two corresponding groups. (Not for HP, NC, or CH due to low numbers of studies.)
(0.08 MB DOC)

Table S7 Sensitivity analyses for the association of the *INSIG2* SNP with BMI regarding sex, age, published status, or self-reported or measured BMI. Stated values are beta-estimates (p-values) based on fixed and random effects models, I^2^ (p-value of Q test) for each group, and p-values testing for difference between the beta-estimates of the two groups. (Not for HP, All-NC, or All-CH due to low numbers of studies.)
(0.07 MB DOC)

Text S1 References of included published studies; data form sent to each study partner; predefined analysis plan; amendment to analysis plan; and classification of studies due by study type.
(0.18 MB DOC)

## References

[1] Herbert A, Gerry NP, McQueen MB, Heid IM, Pfeufer A (2006). A common genetic variant is associated with adult and childhood obesity.. Science.

[2] Yabe D, Brown MS, Goldstein JL (2002). Insig-2, a second endoplasmic reticulum protein that binds SCAP and blocks export of sterol regulatory element-binding proteins.. Proc Natl Acad Sci U S A.

[3] Yabe D, Komuro R, Liang G, Goldstein JL, Brown MS (2003). Liver-specific mRNA for Insig-2 down-regulated by insulin: implications for fatty acid synthesis.. Proc Natl Acad Sci U S A.

[4] Dina C, Meyre D, Samson C, Tichet J, Marre M (2007). Comment on “A common genetic variant is associated with adult and childhood obesity”.. Science.

[5] Loos RJ, Barroso I, O'Rahilly S, Wareham NJ (2007). Comment on “A common genetic variant is associated with adult and childhood obesity”.. Science.

[6] Rosskopf D, Bornhorst A, Rimmbach C, Schwahn C, Kayser A (2007). Comment on “A common genetic variant is associated with adult and childhood obesity”.. Science.

[7] Pollex RL, Ban MR, Young TK, Bjerregaard P, Anand SS (2007). Association between the -455T>C promoter polymorphism of the APOC3 gene and the metabolic syndrome in a multi-ethnic sample.. BMC Med Genet.

[8] Ciullo M, Nutile T, Dalmasso C, Sorice R, Bellenguez C (2008). Identification and replication of a novel obesity locus on chromosome 1q24 in isolated populations of Cilento.. Diabetes.

[9] Kumar J, Sunkishala RR, Karthikeyan G, Sengupta S (2007). The common genetic variant upstream of INSIG2 gene is not associated with obesity in Indian population.. Clin Genet.

[10] Smith AJ, Cooper JA, Li LK, Humphries SE (2007). INSIG2 gene polymorphism is not associated with obesity in Caucasian, Afro-Caribbean and Indian subjects.. Int J Obes (Lond).

[11] Boes E, Kollerits B, Heid IM, Hunt SC, Pichler M (2008). INSIG2 Polymorphism Is Neither Associated With BMI Nor With Phenotypes of Lipoprotein Metabolism.. Obesity (Silver Spring).

[12] Lyon HN, Emilsson V, Hinney A, Heid IM, Lasky-Su J (2007). The association of a SNP upstream of INSIG2 with body mass index is reproduced in several but not all cohorts.. PLoS Genet.

[13] Heid IM, Vollmert C, Hinney A, Doering A, Geller F (2005). Association of the 103I *MC4R* Allele with Decreased Body Mass in 7937 Participants of Two Population-Based Surveys.. J Med Genet.

[14] Hill JO, Wyatt HR, Reed GW, Peters JC (2003). Obesity and the environment: where do we go from here?. Science.

[15] Hill JO, Peters JC (1998). Environmental contributions to the obesity epidemic.. Science.

[16] Monsees GM, Tamimi RMK, Kraft P (2009). Genome-wide association scans for secondary traits using case-control samples.. Genet Epidemiol.

[17] Nebel A, Croucher PJ, Stiegeler R, Nikolaus S, Krawczak M (2005). No association between microsomal triglyceride transfer protein (MTP) haplotype and longevity in humans.. Proc Natl Acad Sci U S A.

[18] Marvelle AF, Lange LA, Qin L, Adair LS, Mohlke KL (2008). Association of FTO with obesity-related traits in the Cebu Longitudinal Health and Nutrition Survey (CLHNS) cohort.. Diabetes.

[19] Loefgen E, Pouta A, Wendt LV, Tapanainen J, Isojaervi JI (2008). Epilepsy in the northern Finnish birth cohort 1966 with special reference to fertility.. Epilepsy Behav.

[20] Jaervelin MR, Sovio U, Kind V, Lauren L, Xu B (2004). Early Life Factor and Blood Pressure at Age 31 Years n the 1966 Northern Finland Birth Cohort.. Hypertension.

[21] Andreasen CH, Mogensen MS, Borch-Johnsen K, Sandbaek A, Lauritzen T (2008). Non-replication of genome-wide based associations between common variants in INSIG2 and PFKP and Obesity Studies of 18,014 Danes.. PloS ONE.

[22] Liu YJ, Liu XG, Wang L, Dina C, Yan H (2008). Genome-wide association scans identified CTNNBL1 as a novel gene for obesity.. Hum Mol Genet.

[23] Zhao Y, Chan MY, Zhou S, Heng CK (2008). Effects of atherogenic diet and atorvastatin treatment on gene expression profiles in the C57BL/6J mouse liver.. Gene Expr.

[24] Koch A, Konig B, Spielmann J, Leitner A, Stangl GI (2007). Thermally oxidized oil increases the expression of insulin-induced genes and inhibits activation of sterol regulatory element-binding protein-2 in rat liver.. J Nutr.

[25] Reinehr T, Hinney A, Nguyen TT, Hebebrand J (2008). Evidence of an influence of a polymorphism near the INSIG2 on weight loss during a lifestyle intervention in obese children and adolescents.. Diabetes.

[26] Reinehr T, Hinney A, Toschke AM, Hebebrand J (2009). Aggravating effect of INSIG2 and FTO on overweight reduction in a one-year lifestyle intervention.. Arch Dis Child.

[27] Orkunoglu-Suer FE, Gordish-Dressman H, Clarkson PM, Thompson PD, Angelopoulos TJ (2008). *INSIG2* gene polymorphism is associated with increased subcutaneous fat in women and poor response to resistance training in men.. BMC Med Genet.

[28] Hinney A, Nguyen TT, Scherag A, Friedel S, Bronner G (2007). Genome wide association (GWA) study for early onset extreme obesity supports the role of fat mass and obesity associated gene (FTO) variants.. PLoS ONE.

[29] Heid IM, Wagner SA, Gohlke H, Iglseder B, Mueller JC (2006). Genetic architecture of the APM1 gene and its influence on adiponectin plasma levels and parameters of the metabolic syndrome in 1,727 healthy caucasians.. Diabetes.

[30] DerSimonian R, Laird N (1986). Meta-analysis in clinical trials.. Control Clin Trials.

