# Supplementary figures and images for: Meta-Analysis of the INSIG2 Association with Obesity Including 74,345 Individuals: Does Heterogeneity of Estimates Relate to Study Design?
Source: PLoS Genet. 2009 Oct 23;5(10):e1000694. doi: 10.1371/journal.pgen.1000694 (PMC2757909; doi:10.1371/journal.pgen.1000694)

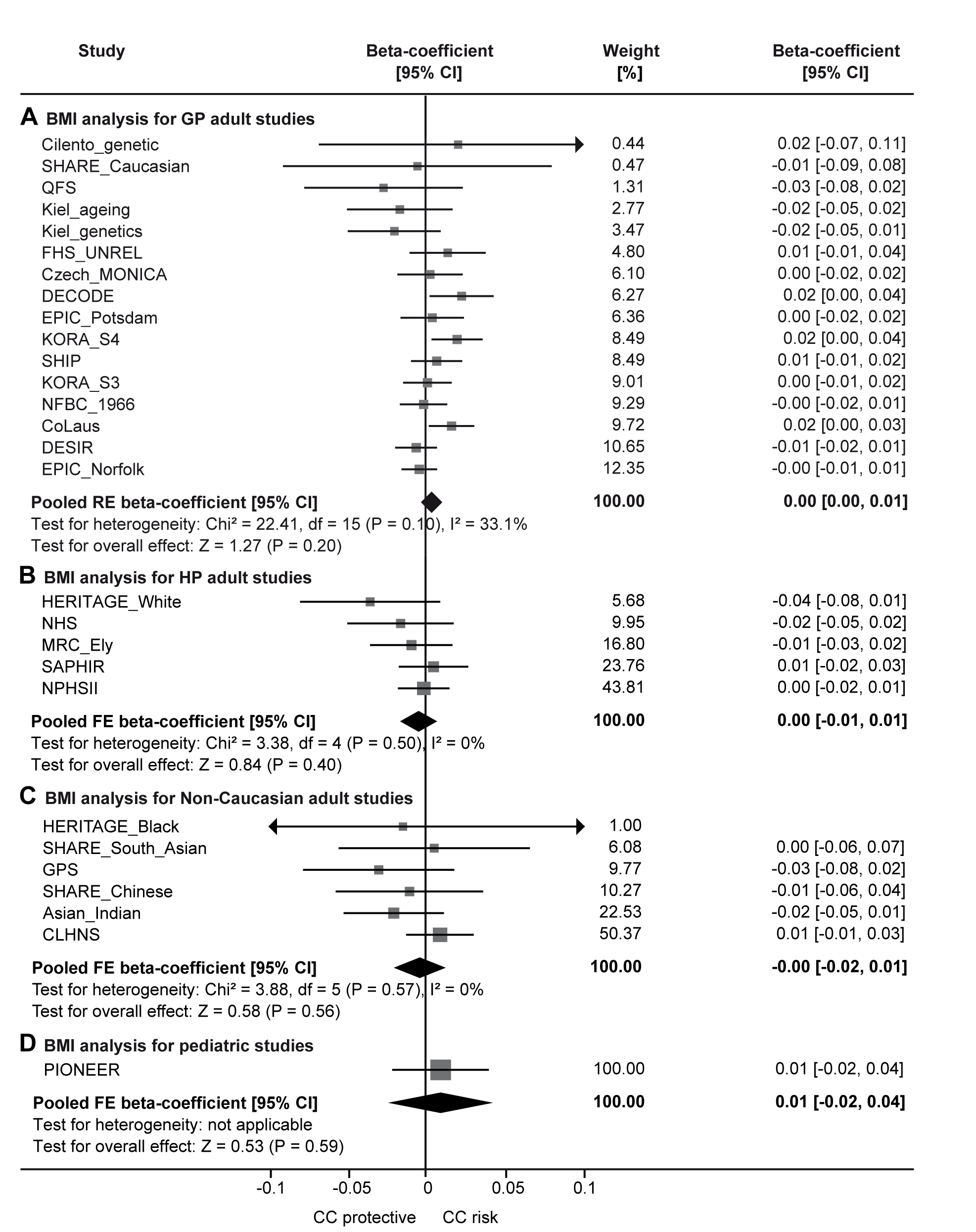

Supplement: Figure S1 — Forest plot on estimates of the INSIG2 rs7566605 association with BMI. Association of the INSIG2 SNP with BMI for (A) the Caucasian adult general-population based studies (GP), (B) the Caucasian adult studies selected for healthy population (HP), (C) the Non-Caucasian adult studies, (D) the pediatric studies. Shown are recessive model beta estimates comparing the CC genotype versus CG or GG for each study and pooled estimates. The fixed effect (FE) model beta is shown in case of no significant heterogeneity as tested by the Q-statistics; a random effect (RE) model is shown otherwise. (0.44 MB TIF) [file pgen.1000694.s001.tif]
