# Supplementary material for: Meta-Analysis of the INSIG2 Association with Obesity Including 74,345 Individuals: Does Heterogeneity of Estimates Relate to Study Design?
Source: PLoS Genet. 2009 Oct 23;5(10):e1000694. doi: 10.1371/journal.pgen.1000694 (PMC2757909; doi:10.1371/journal.pgen.1000694)
Supplement: Table S1 — Characterization of eligible and recruited studies. (0.12 MB DOC) [file pgen.1000694.s002.doc]

***Table S1: Characterization of included studies***

| Study Abbreviation | Pop | Design | Natio-nality | <year 2000 | # subjects | Obese [%] | CC  [%] | MAF [%] | Call rate  [%] | P HWE |
| --- | --- | --- | --- | --- | --- | --- | --- | --- | --- | --- |
| ***General Population (GP) Caucasian adults*** | | | | | | | | | | |
| Cilento_genetic | 1 | 1 | 10 | 2 | 384 | 18.49 | 4.95 | 23.70 | 98.8 | 0.572 |
| CoLaus | 1 | 1 | 11 | 2 | 5463 | 15.76 | 10.96 | 32.99 | 95.0 | 0.784 |
| Czech_MONICA | 1 | 1 | 12 | 1 | 2498 | 29.34 | 10.73 | 32.83 | 96.2 | 0.932 |
| DECODE | 1 | 1 | 13 | 2 | 5187 | 35.05 | 11.68 | 34.04 | 97.3 | 0.748 |
| DESIR | 1 | 1 | 14 | 1 | 4904 | 9.42 | 10.62 | 32.41 | 98.0 | 0.716 |
| EPIC_Norfolk | 1 | 1 | 15 | 1 | 6732 | 13.35 | 10.09 | 31.92 | 98.7 | 0.722 |
| EPIC_Potsdam | 1 | 1 | 16 | 1 | 2453 | 16.39 | 10.31 | 32.21 | 98.1 | 0.927 |
| FHS_unrel | 1 | 1 | 17 | 1 | 1491 | 11.27 | 11.54 | 32.49 | 99.0 | 0.553 |
| Kiel_ageing | 1 | 1 | 16 | 2 | 890 | 4.38 | 10.34 | 31.85 | 99.5 | 0.814 |
| Kiel_genetics | 1 | 1 | 16 | 2 | 1065 | 14.65 | 10.61 | 32.91 | 99.5 | 0.778 |
| KORA_S3 | 1 | 1 | 16 | 1 | 4087 | 20.80 | 10.74 | 33.64 | 99.3 | 0.107 |
| KORA_S4 | 1 | 1 | 16 | 2 | 3996 | 23.40 | 10.86 | 33.75 | 97.9 | 0.146 |
| NFBC 1966 | 1 | 1 | 18 | 1 | 4452 | 9.25 | 11.73 | 34.22 | 99.7 | 0.974 |
| QFS | 1 | 3 | 19 | 1 | 844 | 27.25 | 10.43 | 30.57 | 95.9 | 0.139 |
| SHARE_Caucasian | 1 | 1 | 114 | 2 | 232 | 27.16 | 6.90 | 30.60 | 98.0 | 0.095 |
| SHIP | 1 | 1 | 16 | 1 | 4166 | 25.52 | 9.91 | 32.06 | 98.9 | 0.309 |
| ***Healthy population (HP) Caucasian adults*** | | | | | | | | | | |
| HERITAGE_White | 2 | 3 | 17+19 | 1 | 497 | 19.52 | 7.44 | 28.77 | 99.0 | 0.446 |
| MRC_Ely | 2 | 1 | 15 | 2 | 1683 | 22.16 | 10.70 | 31.79 | 97.9 | 0.260 |
| NHS | 2 | 1 | 17 | 1 | 1046 | 13.29 | 11.85 | 34.32 | 95.3 | 0.945 |
| NPHSII | 2 | 1 | 1? | 1 | 2718 | 13.87 | 10.45 | 31.31 | 98.1 | 0.122 |
| SAPHIR | 2 | 1 | 111 | 2 | 1696 | 18.93 | 12.09 | 34.79 | 98.3 | 1.000 |
| ***Obesity case-control studies (OB) with Caucasian adults*** | | | | | | | | | | |
| American_Polish | 3 | 2 | 17+112 | 2 | 2757 | 62.86 | 10.63 | 33.01 | 96.1 | 0.061 |
| Essen_obese | 3 | 2 | 16 | 2 | 1379 | 71.65 | 10.88 | 33.18 | 100.0 | 0.156 |
| OBENUTIC | 3 | 2 | 113 | 2 | 1135 | 34.89 | 11.28 | 32.86 | 96.0 | 0.563 |
| OB_adult | 3 | 2 | 14 | 1 | 1515 | 68.91 | 12.08 | 35.48 | 96.0 | 0.484 |
| Swiss_obese | 3 | 2 | 11 | 2 | 1327 | 53.50 | 12.43 | 33.76 | 97.4 | 1.000 |
| Utah_obese | 3 | 2 | 17 | 2 | 1616 | 63.43 | 10.95 | 32.61 | 98.3 | 0.196 |
| ***Other ethnicities (all adults)*** | | | | | | | | | |  |
| Asian_Indian | 1 | 1 | 3 | 2 | 1536 | 5.73 | 9.38 | 30.27 | 96.5 | 0.715 |
| CLHNS | 1 | 1 | 5 | 2 | 1747 | 9.39 | 20.09 | 44.99 | 98.0 | 0.801 |
| GPS | 1 | 1 | 4 | 2 | 1284 | 19.78 | 4.52 | 20.37 | 98.0 | 0.436 |
| SHARE_Chinese^a^ | 1 | 1 | 7 | 2 | 301 | 5.65 | 12.96 | 36.05 | 98.0 | 1.000 |
| SHARE_South_Asian | 1 | 1 | 6 | 2 | 322 | 14.60 | 7.76 | 25.62 | 98.0 | 0.242 |
| HERITAGE_Black ^a^ | 2 | 3 | 2 | 1 | 274 | 31.75 | 2.55 | 26.28 | 99.0 | <0.0001 |
| ***Children (all Caucasian)*** | | | | | | | | | |  |
| CHOBES | 3 | 2 | 14 | 1 | 1367 | 66.20 | 8.56 | 30.61 | 98.7 | 0.183 |
| Essen_trios | 3 | 3 | 16 | 1 | 861 | 100.00 | 12.31 | 34.38 | 98.8 | 0.553 |
| PIONEER | 1 | 1 | 16 | 2 | 1015 | 3.55 | 10.25 | 32.81 | 96.4 | 0.477 |
|  |  |  |  |  |  |  |  |  |  |  |
| *Total* |  |  |  |  | 74,345 ^a^ |  |  |  |  |  |

**Pop** (population): 1 = general (GP), 2 = healthy (HP), 3 = obesity study (OB); **Design**: 1 = cross-sectional, 2 = case-control, 3 = families; **Nationality**: 10 = Italian, 11 = Swiss, 12 = Czech, 13 = Icelandic, 14 = French, 15 = UK, 16 = German, 17 = US, 18 = Finnish, 19 = French-Canadian, 111 = Austrian, 112 = Polish, 113 = Spanish, 2 = African American, 114 = European, 3 = Indian, 4 = Inuit, 5 = Philippine, 6 = South Asian, 7 = Chinese; **< year 2000**: year of assessing the obesity status was prior to the year 2000: 1 = yes, 2 = no; # **subjects** = number of subjects; **Obese [%]** = percentage of obese subjects as defined by BMI ≥30 kg/m²; **CC [%]** = percentage of subjects with CC genotype.

**MAF** = minor allele frequency

**P HWE** = p-value of exact test for Hardy-Weinberg equilibrium (HWE), using 10,000 Monte Carlo permutations; the test was conducted in all subjects for GP and HP studies, only in non-obese subjects for OB studies.

^a^ Were eligible according to defined criteria, sent data, were included for pooling BMI beta-estimates, but were not included for pooling obesity ORs due to <3 obese subjects with CC genotype and are not included in ‘total’ number of subjects.
