# Supplementary material for: Meta-Analysis of the INSIG2 Association with Obesity Including 74,345 Individuals: Does Heterogeneity of Estimates Relate to Study Design?
Source: PLoS Genet. 2009 Oct 23;5(10):e1000694. doi: 10.1371/journal.pgen.1000694 (PMC2757909; doi:10.1371/journal.pgen.1000694)
Supplement: Table S2 — More details on study characteristics. (0.20 MB DOC) [file pgen.1000694.s003.doc]

**Table S2**: More details on study characteristics.

| Study name abbreviation | Study name | Pub-lished^a^ | Reference^b^ | Male [%]^c^ | Mean age ± SE [years] ^c^ | Mean BMI ± SE [kg/m²] ^c^ |  |
| --- | --- | --- | --- | --- | --- | --- | --- |
| ***General Population (GP) Caucasian adults*** | | | | | | | |
| Cilento_genetic | Genetic study of complex diseases in the isolated populations of the Cilento and Vallo di Diano National Park | 1 | [1] | 49.7 | 53.8±18.3 | 26.7±4.5 |  |
| CoLaus | The Lausanne Population based Study | 2 |  | 47.6 | 53.2±10.8 | 25.8±4.6 |  |
| Czech_MONICA | Czech MONICA study | 2 |  | 46.6 | 48.8±10.7 | 27.9±4.9 |  |
| DECODE | deCODE's obesity genetic study | 1 | [2] | 46.0 | 54.4±16.6 | 28.7±6.7 |  |
| DESIR | Données Epidémiologiques sur le Syndrome d’Insulino-Résistance cohort | 1 | [3] | 49.7 | 47.3±10.0 | 24.7±3.8 |  |
| EPIC_Norfolk | European Prospective Investigation of Cancer (EPIC)-Norfolk | 1 | [4] | 45.5 | 57.8±9.3 | 26.1±3.7 |  |
| EPIC_Potsdam | European Prospective Investigation into Cancer and Nutrition Potsdam | 2 |  | 38.9 | 49.8±9.0 | 26.1±4.3 |  |
| FHS_unrel | FHS unrelated | 1 | [2] | 52.3 | 37.1±8.9 | 25.1±4.2 |  |
| Kiel_ageing | Kiel Genetics of Healthy Ageing Study | 2 | [5] | 26.7 | 98.5±2.7 | 22.9±3.6 |  |
| Kiel_genetics | Kiel Genetics of Healthy Ageing Study | 2 | [5] | 25.7 | 66.9±4.1 | 26.0±4.0 |  |
| KORA_S3 | KORA Survey S3 | 1 | [2] | 50.1 | 48.7±13.8 | 26.9±4.5 |  |
| KORA_S4 | KORA Survey S4 | 1 | [6] | 49.7 | 49.3±13.9 | 27.2±4.7 |  |
| NFBC 1966 | North Finnish Birth Cohort 1966 | 2 | [7,8] | 50.0 | 31 ^d^ | 24.7±4.2 |  |
| QFS | Québec Family Study | 2 |  | 43.7 | 42.7±16.6 | 27.7±7.6 |  |
| SHARE_Caucasian | Study of Health Assessment and Risk in Ethnic Groups - Caucasian | 1 | [9] | 46.1 | 51.1±11.0 | 27.4±4.6 |  |
| SHIP | Study of Health in Pomerania | 1 | [10] | 49.5 | 49.8±16.4 | 27.3±4.8 |  |
|  |  |  |  |  |  |  |  |
| ***Healthy population (HP) Caucasian adults*** | | | | | | | |
| HERITAGE_White | HERITAGE Family Study White | 2 |  | 48.1 | 35.7±14.5 | 25.9±5.0 |  |
| MRC_Ely | Medical Research Council- Ely Study | 1 | [4] | 54.1 | 61.1±9.1 | 27.2±4.8 |  |
| NHS | The Nurses' health Study | 1 | [6] | 0 | 42.6±6.9 | 25.0±4.9 |  |
| NPHSII | Second Northwick Park Heart Study | 1 | [11] | 100.0 | 56.1±3.4 | 26.5±3.5 |  |
| SAPHIR | SAPHIR-Study | 1 | [12] | 63.1 | 51.8±6.1 | 26.8±4.1 |  |
|  |  |  |  |  |  |  |  |
| ***Obesity case-control studies (OB) with Caucasian adults*** | | | | | | | |
| American_Polish | American_Polish | 1 | [6] | 42.6 51.1 | 56.0±8.9 57.5± 9.3 | 35.4± 3.0 22.2±2.1 |  |
| Essen_obese | Essen obesity case-control study | 1 | [2] | 36.8 37.6 | 46.3±14.7 25.5±4.9 | 36.0±5.4 18.2±1.0 |  |
| OBENUTIC | Obesity, nutrition and gene-diet interactions | 2 |  | 32.6 33.4 | 48.1±13.7 47.1±13.4 | 36.5±6.8 24.6±2.9 |  |
| OB_adult | OB adult | 1 | [3] | 28.8 42.7 | 45.8±12.2 53.0±15.4 | 43.2±9.1 24.4±3.0 |  |
| Swiss_obese^e^ | Genetics and Pharmacogenomics of Obesity | 2 | [13] | 21.7 24.3 | 41.4±10.5 40.1±9.3 | 43.8±5.9 23.5±2.9 |  |
| Utah_obese | Utah Obesity Study | 1 | [12] | 18.6 46.5 | 44.3±11.4 52.6±8.7 | 46.0±7.6 25.2±2.9 |  |
|  |  |  |  |  |  |  |  |

| ***Other ethnicities (all adults)*** | | | | | | | |
| --- | --- | --- | --- | --- | --- | --- | --- |
| Asian_Indian | Indian Study on INSIG2 | 1 | [14] | 57.8 | 38.7±14.1 | 22.6±4.3 |  |
| CLHNS | Cebu Longitudinal Health and Nutrition Survey | 2 | [15] | 0.0 | 48.5±6.1 | 24.3±4.4 |  |
| GPS | Greenland Population Study | 1 | [9] | 44.2 | 43.5±14.2 | 26.2±5.0 |  |
| SHARE_Chinese ^f^ | Study of Health Assessment and Risk in Ethnic Groups – Chinese | 1 | [9] | 51.2 | 47.9±9.0 | 24.0±3.6 |  |
| SHARE_South_Asian | Study of Health Assessment and Risk in Ethnic Groups – South Asian | 1 | [9] | 54.3 | 49.5±9.3 | 26.3±4.1 |  |
| HERITAGE_Black ^f^ | HERITAGE Family Study Black | 2 |  | 32.8 | 33.7±11.7 | 27.9±6.0 |  |
|  |  |  |  |  |  |  |  |
| ***Children (all Caucasian)*** | | | | | | | |
| CHOBES | Childhood Obesity | 1 | [3] | 47.0 | 11.9±0.2 | 21.1±0.3 |  |
| Essen_trios | Essen obesity family study | 1 | [6] | 55.4 | 14.1±3.8 | 31.0±6.0 |  |
| PIONEER | Puberty onset - influence in nutritional, environmental and endogenous regulators WP 2 | 2 |  | 0 | 12.3±1.2 | 19.4±3.2 |  |
|  |  |  |  |  |  |  |  |

^a^ at time of recruitment for this meta-analysis. ^b^ Manuscripts published after January 2008 or a study reference regarding other investigations are also included in the reference list. *^c^* If applicable, values for cases and controls are given separately. ^d^ All subjects had the same age at BMI assessment. ^e^ Cases from Klinik Lindberg, Winterthur, Switzerland, controls by University Kiel, Germany, ^f^ Were eligible according to defined criteria, sent data, were included for pooling BMI beta-estimates, but were not included for pooling obesity ORs due to <3 obese subjects with CC genotype.
