# Supplementary material for: Meta-Analysis of the INSIG2 Association with Obesity Including 74,345 Individuals: Does Heterogeneity of Estimates Relate to Study Design?
Source: PLoS Genet. 2009 Oct 23;5(10):e1000694. doi: 10.1371/journal.pgen.1000694 (PMC2757909; doi:10.1371/journal.pgen.1000694)
Supplement: Table S3 — Stronger genetic effects when comparing more extreme degrees of obesity as an explanation of the heterogeneity of the INSIG2 rs7566605 association (Hypothesis 2). Pooled association estimates for increasing degrees of obesity for all Caucasian adult studies combined (All-CA) as well as stratified by study type (GP = general population, HP = healthy population, OB = obesity study), for all Non-Caucasian studies (All-NC), but not for children studies due to non-comparability of BMI categories. Numbers stated are ORs comparing cases versus controls (p-values) from the pooled analysis (fixed and random effects), number of cases/controls (number of pooled studies), and the I2 (p-value from Q statistics). (0.07 MB DOC) [file pgen.1000694.s004.doc]

**Table S3: Stronger genetic effects when comparing more extreme degrees of obesity as a potential explanation of the heterogeneity of the *INSIG2* rs7566605 association with obesity (hypothesis 2).** Pooled association estimates for increasing degrees of obesity for all Caucasian adult studies combined (All-CA) as well as stratified by study type (GP=general population, HP=healthy population, OB=obesity study), for all Non-Caucasian studies (All-NC), but not for children studies due to non-comparability of BMI categories. Numbers stated are ORs comparing ‘cases’ versus ‘controls’ (p-values) from the pooled analysis (fixed and random effects), number of cases/controls (number of pooled studies), and the I² (p-value from Q statistics).

|  | BMI cut-off [kg/m²] for ‘controls’ |  | BMI cut-off [kg/m²] for ‘cases’ .  30.0 32.5 35.0 37.5 40.0 | | | | |
| --- | --- | --- | --- | --- | --- | --- | --- |
| All-CA | 30.0 | **OR** (p-value) fixed effect **OR** (p-value) random effect # cases/controls (studies)  Heterogeneity I² (p-value) | **1.071** (0.040) **1.050** (0.271) 13,195/48,266 (25)  34.8 (0.046) | **1.132** (0.002)  **1.105** (0.064) 8545/47,124 (22) 37.4 (0.040) | **1.130** (0.015)  **1.113** (0.078) 5506/46,700 (21) 23.0 (0.167) | **1.142** (0.026)  **1.142** (0.035) 4002/45,791 (20) 6.8 (0.372) | **1.210** (0.011)  **1.227** (0.023) 2884/31,363 (14) 19.9 (0.237) |
|  | 25.0 | **OR** (p-value) fixed effect **OR** (p-value) random effect # cases/controls (studies) Heterogeneity I² (p-value) | **1.086** (0.029)  **1.074** (0.101) 14,352/26,116 (26)  18.8 (0.196) | **1.156** (0.001)  **1.141** (0.007) 9694/25,257 (23)  15.0 (0.257) | **1.183** (0.002)  **1.175** (0.005) 6383/24,931 (22)  5.4 (0.387) | **1.221** (0.002)  **1.221** (0.002) 4430/24,446 (21)  0.0 (0.582) | **1.265** (0.003)  **1.265** (0.006) 3054/15,953 (15)  8.9 (0.353) |
|  | 20.0 | **OR** (p-value) fixed effect **OR** (p-value) random effect # cases/controls (studies) Heterogeneity I² (p-value) | **1.081** (0.241)  **1.081** (0.329) 13,061/2885 (22)  0.0 (0.461) | **1.145** (0.098)  **1.145** (0.105) 8961/3051 (22)  0.0 (0.736) | **1.158** (0.077)  **1.157** (0.122) 5957/3051 (22)  0.4 (0.453) | **1.162** (0.166) **1.136** (0.303) 4295/3004 (21)  20.8 (0.192) | **1.285** (0.088) **1.213**(0.333) 3075/1946 (15) 52.8 (0.008) |
| GP | 30.0 | **OR** (p-value) fixed effect **OR** (p-value) random effect # cases/controls (studies) Heterogeneity I² (p-value) | **1.097** (0.015) **1.092** (0.035) 9162/39,682 (16)  10.9 (0.329) | **1.196** (0.0002)  **1.189** (0.001) 4794/38,349 (13) 15.4 (0.289) | **1.215** (0.002)  **1.215** (0.002) 2527/38,349 (13) 0.0 (0.534) | **1.220** (0.010)  **1.220** (0.011) 1421/37,440 (12) 1.5 (0.429) | **1.380** (0.004)  **1.387** (0.008) 763/27,635 (9) 8.0 (0.369) |
|  | 25.0 | **OR** (p-value) fixed effect **OR** (p-value) random effect # cases/controls (studies) Heterogeneity I² (p-value) | **1.092** (0.048)  **1.087** (0.079) 9162/21,041 (16)  8.6 (0.355) | **1.198** (0.001)  **1.198** (0.001) 4794/20,161 (13)  0.0 (0.532) | **1.257** (0.001)  **1.257** (0.001) 2527/20,161 (13)  0.0 (0.640) | **1.313** (0.002)  **1.313** (0.002) 1421/19,676 (12)  0.0 (0.532) | **1.414** (0.003)  **1.410** (0.013) 763/13,397 (9)  18.5 (0.278) |
|  | 20.0 | **OR** (p-value) fixed effect **OR** (p-value) random effect # cases/controls (studies) Heterogeneity I² (p-value) | **1.116** (0.241)  **1.116** (0.240) 8616/2101 (13)  0.0 (0.837) | **1.173** (0.098)  **1.173** (0.098) 4794/2279 (13)  0.0 (0.760) | **1.215** (0.077)  **1.215** (0.077) 2527/2279 (13)  0.1 (0.445) | **1.192** (0.166) **1.159** (0.399) 1421/2232 (12)  40.6 (0.070) | **1.312** (0.088) **1.225** (0.506) 763/1360 (9) 66.8 (0.002) |
| HP | **30.0** | **OR** (p-value) fixed effect **OR** (p-value) random effect # cases/controls (studies) Heterogeneity I² (p-value) | **0.796** (0.028) **0.796** (0.028) 1307/6333 (5)  0.0 (0.415) | **0.789** (0.113)  **0.789** (0.113) 577/5933 (4) 0.0 (0.942) | **0.880** (0.535)  **0.880** (0.535) 277/5933 (4) 0.0 (0.693) | **1.038** (0.888)  **1.038** (0.888) 144/5933 (4) 0.0 (0.966) | **1.415** (0.486)  **1.415** (0.486) 31/1310 (1) - |
|  | **25.0** | **OR** (p-value) fixed effect **OR** (p-value) random effect # cases/controls (studies) Heterogeneity I² (p-value) | **0.865** (0.207)  **0.865** (0.207) 1307/3045 (5)  0.0 (0.456) | **0.856** (0.327)  **0.856** (0.327) 577/2797 (4)  0.0 (0.894) | **0.959** (0.843)  **0.959** (0.846) 277/2797 (4)  0.0 (0.660) | **1.139** (0.629)  **1.139** (0.629) 144/2797 (4)  0.0 (0.941) | **1.604** (0.355)  **1.604** (0.355) 31/583 (1)  - |
| OB | **30.0** | **OR** (p-value) fixed effect **OR** (p-value) random effect # cases/controls (studies) Heterogeneity I² (p-value) | **1.183** (0.076)^a^ **1.187** (0.221) ^a^ 2726/2251 (4)  53.3 (0.092) | **1.063** (0.491) 1.080 (0.617) 3174/2842 (5) 64.7 (0.023) | **1.000** (0.999)  **0.983** (0.920) 2702/2418 (4) 66.8 (0.029) | **1.035**(0.727)  **1.028** (0.863) 2437/2418 (4) 57.8 (0.068) | **1.075** (0.486)  **1.077** (0.577) 2090/2418 (4) 36.8 (0.191) |
|  | **25.0** | **OR** (p-value) fixed effect **OR** (p-value) random effect # cases/controls (studies) Heterogeneity I² (p-value) | **1.227** (0.027)  **1.221** (0.055) 3883/2030 (5)  20.9 (0.281) | **1.154** (0.105)  **1.134** (0.334) 4323/2299 (6)  51.4 (0.067) | **1.097** (0.339)  **1.055** (0.712) 3579/1973 (5)  53.2 (0.073) | **1.113** (0.307)  **1.093** (0.501) 2865/1973 (5)  35.9 (0.187) | **1.125** (0.298)  **1.125** (0.230) 2260/1973 (5)  0.0 (0.503) |
|  | **20.0** | **OR** (p-value) fixed effect **OR** (p-value) random effect # cases/controls (studies) Heterogeneity I² (p-value) | **1.258** (0.267)  **1.156** (0.616) 3138/511 (4)  41.7 (0.162) | **1.240** (0.291)  **1.164** (0.549) 3590/540 (5)  28.8 (0.229) | **1.140** (0.532)  **1.093** (0.726) 3153/540 (5)  24.0 (0.261) | **1.146** (0.543) **1.097** (0.728) 2730/540 (5)  26.6 (0.244) | **1.235** (0.363) **1.187** (0.537) 2281/540 (5) 28.0 (0.235) |

^a^ Differs slightly from main OB analysis as studies are excluded here if they do not have cases in the range of 30.0–32.5 kg/m² or controls with 25.0–30.0 kg/m².
