# Supplementary material for: Meta-Analysis of the INSIG2 Association with Obesity Including 74,345 Individuals: Does Heterogeneity of Estimates Relate to Study Design?
Source: PLoS Genet. 2009 Oct 23;5(10):e1000694. doi: 10.1371/journal.pgen.1000694 (PMC2757909; doi:10.1371/journal.pgen.1000694)
Supplement: Table S4 — Sensitivity analyses for the association of the INSIG2 SNP with obesity regarding sex, age, published or unpublished status of studies, self-reported or measured BMI. Stated values are ORs (p-values) based on fixed or random effects models, the I2 (p-value of Q test) for each group, and p-values testing for difference between the fixed effect [random effect] ORs of the two corresponding groups. (Not for HP, OB, All-NC, or All-CH due to low numbers of studies.) (0.07 MB DOC) [file pgen.1000694.s005.doc]

**Table S4: Sensitivity analyses for the association of the *INSIG2* SNP with obesity regarding sex, age, published or unpublished status of studies, self-reported or measured BMI.** Stated values are ORs (p-values) based on fixed or random effects models, the I² (p-value of Q test) for each group, and p-values testing for difference between the fixed effect [random effect] ORs of the two corresponding groups. (Not for HP, OB, All-NC, or All-CH due to low numbers of studies.)

| Group |  | # subjects (# studies) | OR (p-value)  fixed effect | OR (p-value)  random effect | I² (p-value) | Testing for difference p-value^e^ |
| --- | --- | --- | --- | --- | --- | --- |
| Men^a^ | All-CA | 58,930 (25) | 1.054 (0.272) | 1.054 (0.272) | 0.0 (0.596) |  |
|  | GP | 22,650 (15) | 1.079 (0.175) | 1.079 (0.175) | 0.0 (0.777) |  |
| Women^a^ | All-CA | 64,838 (25) | 1.110 (0.016) | 1.092 (0.092) | 24.4 (0.133) | 0.420 [0.615] |
|  | GP | 25,956 (16) | 1.126 (0.022) | 1.116 (0.060) | 14.5 (0.288) | 0.571 [0.676] |
| Age ≥ 50 yrs^b^ | All-CA | 32,941 (25) | 0.091 (0.032) | 0.063 (0.292) | 38.9 (0.026) |  |
|  | GP | 24,459 (15) | 1.111 (0.037) | 1.095 (0.188) | 34.7 (0.091) |  |
| Age < 50 yrs^b^ | All-CA | 29,029 (24) | 1.074 (0.140) | 1.055 (0.381) | 29.1 (0.092) | 0.762 [0.910] |
|  | GP | 24,385 (14) | 1.090 (0.133) | 1.090 (0.133) | 0.0 (0.632) | 0.802 [0.964] |
| Published studies^c^ | All-CA | 42,277 (15) | 1.100 (0.017) | 1.080 (0.193) | 45.7 (0.028) |  |
|  | GP | 31,179 (9) | 1.120 (0.014) | 1.114 (0.078) | 33.9 (0.147) |  |
| Unpublished studies^c^ | All-CA | 23,936 (12) | 1.033 (0.555) | 1.008 (0.918) | 36.9 (0.095) | 0.351 [0.461] |
|  | GP | 17,665 (7) | 1.047 (0.496) | 1.047 (0.496) | 0.0 (0.669) | 0.410 [0.501] |
| Self-reported BMI^d^ | All-CA | 3001 (3) | 0.814 (0.302) | 0.814 (0.302) | 0.0 (0.534) |  |
|  | GP | 1955 (2) | 0.816 (0.447) | 0.839 (0.572) | 20.3 (0.263) |  |
| Measured BMI^d^ | All-CA | 63,212 (24) | 1.084 (0.013) | 1.063 (0.187) | 43.7 (0.012) | 0.156 [0.192] |
|  | GP | 46,889 (14) | 1.104 (0.011) | 1.100 (0.021) | 9.3 (0.351) | 0.264 [0.387] |

^a^ Combined results of study-specific analyses stratified for men or women. ^b^ Combined results of study-specific analyses stratified for subjects ≥ 50 years or < 50 years of age. ^c^ Combined results for the published or the unpublished studies. ^d^ Combined results for the studies with self-reported BMI or with measured BMI.
^e^ Testing for difference of fixed effect [random effects] OR estimates. OR = Odds Ratio, vs. = versus, All-CA = Caucasian adult studies, GP = general population-based studies.
