# Supplementary material for: Meta-Analysis of the INSIG2 Association with Obesity Including 74,345 Individuals: Does Heterogeneity of Estimates Relate to Study Design?
Source: PLoS Genet. 2009 Oct 23;5(10):e1000694. doi: 10.1371/journal.pgen.1000694 (PMC2757909; doi:10.1371/journal.pgen.1000694)
Supplement: Table S5 — Main results of pooled association of the INSIG2 SNP with body-mass-index (BMI). The analyses for all Caucasian adult studies combined (All-CA) as well as stratified by study type (GP = general population, HP = healthy population, OB = obesity study), for all Non-Caucasian studies (All-NC), and for the children studies (All-CH) indicated some difference between GP and HP studies (Hypothesis 1). Numbers stated are recessive model beta-estimates (p-values), i.e., mean difference of BMI between subjects with the CC genotype compared to subjects with the CG or GG genotype, using fixed or random effects models, the I2 (p-value of Q-statistics), and p-values testing for pair-wise difference between GP, HP, or OB studies. (0.04 MB DOC) [file pgen.1000694.s006.doc]

**Table S5: Main results of pooled association of the *INSIG2* SNP with body-mass-index (BMI).** The analyses for all Caucasian adult studies combined (All-CA) as well as stratified by study type (GP = general population, HP = healthy population, OB = obesity study), for all Non-Caucasian studies (All-NC), and for the children studies (All-CH) indicated some difference between GP and HP studies (hypothesis 1). Numbers stated are recessive model beta-estimates (p-values), i.e. mean difference of BMI between subjects with the CC genotype compared to subjects with the CG or GG genotype, using fixed or random effects models, the I² (p-value of Q-statistics), and p-values testing for pair-wise difference between GP, HP, or OB studies.

|  | # subjects  (# studies) | beta (p-value)  fixed effect | beta (p-value)  random effects | I² (p-value) | p-value test for difference of fixed [random] effects betas^b^ |
| --- | --- | --- | --- | --- | --- |
| All-CA | 66,213 (27) | 0.002 (0.275) | 0.002 (0.413) | 27.8 (0.117) |  |
| All-CA^a^ | 33,957(19) | 0.004 (0.178) | 0.003 (0.318) | 18.6 (0.246) |  |
| GP | 48,844 (16) | 0.004 (0.122) | 0.004 (0.203) | 33.1 (0.097) | GP vs HP: 0.169 [0.176] |
| GP^a^ | 29,046(12) | 0.005 (0.098) | 0.005 (0.202) | 18.9 (0.098) | GP^a^ vs HP^a^: 0.315 [0.597] |
| HP | 7640(5) | -0.004 (0.404) | -0.004 (0.403) | 0.0 (0.496) |  |
| HP^a^ | 4911(3) | -0.002 (0.769) | -0.003 (0.729) | 23.6 (0.270) |  |
| OB |  | Not applicable  Not applicable | | | |
| OB^a^ |  |
| ALL-NC | 4889 (4) | -0.003 (0.660) | -0.006 (0.559) | 20.4 (0.287) |  |
| ALL-CH | 1015 (1) | 0.008 (0.594) | 0.008 (0.594) | - |  |

^a^ Excluding studies published before the response letter in *Science* by Herbert et al., December 2006, in which the hypothesis of potential heterogeneity due to study design and a first call for this meta-analysis were stated (i.e. excluding American_Polish, NHS, KORA_S4, Essen_trios, EPIC_Norfolk, MRC_Ely, DESIR, SHIP, OB_adult). ^b^ Corrected p-values for pair-wise comparison of three subgroups need to be multiplied by three.
