# Supplementary material for: Meta-Analysis of the INSIG2 Association with Obesity Including 74,345 Individuals: Does Heterogeneity of Estimates Relate to Study Design?
Source: PLoS Genet. 2009 Oct 23;5(10):e1000694. doi: 10.1371/journal.pgen.1000694 (PMC2757909; doi:10.1371/journal.pgen.1000694)
Supplement: Table S6 — Exploring potential sources of heterogeneity of the INSIG2 rs7566605 association with BMI (Hypotheses 3–5). Stated values are pooled beta-estimates (p-values) based on fixed or random effects model, I2 (p-values of Q-test) for each group, and p-values testing for difference between the beta-estimates of the two corresponding groups. (Not for HP, NC, or CH due to low numbers of studies.) (0.08 MB DOC) [file pgen.1000694.s007.doc]

**Table S6: Exploring potential sources of heterogeneity of the *INSIG2* rs7566605 association with BMI (hypotheses 3-5).** Stated values are pooled beta-estimates (p-values) based on fixed or random effects model, I² (p-values of Q-test) for each group, and p-values testing for difference between the beta-estimates of the two corresponding groups. (not for HP, NC, or CH due to low numbers of studies)

| Group |  | # subjects  (# studies) | beta (p-value)  fixed effect | beta (p-value)  random effect | I² (p-value) | Testing for difference  p-value ^e^ |
| --- | --- | --- | --- | --- | --- | --- |
| %obese  ≥ 18 %^a^ | All-CA | 25,270 (11) | 0.007 (0.050) | 0.006 (0.156) | 22.9 (0.225) |  |
|  | All-Ca^d^ | 15,425 (8) | 0.004 (0.351) | 0.004 (0.486) | 16.5 (0.300) |  |
|  | GP | 21394 (8) | 0.009 (0.013) | 0.009 (0.019) | 6.3 (0.381) |  |
|  | GP^g^ | 13232 (6) | 0.006 (0.233) | 0.006 (0.247) | 2.9 (0.400) |  |
| %obese  < 18 %^a^ | All-CA | 31214 (10) | -0.000 (0.873) | -0.000 (0.881) | 25.2 (0.211) | 0.101 [0.229] |
|  | All-CA^d^ | 18532 (7) | 0.003 (0.328) | 0.003 (0.564) | 31.7 (0.186) | 0.903 [0.875] |
|  | GP | 27450 (8) | 0.000 (0.967) | 0.000 (0.959) | 37.2 (0.132) | 0.051 [0.101] |
|  | GP^g^ | 15814 (6) | 0.005 (0.244) | 0.003 (0.579) | 40.3 (0.137) | 0.828 [0.691] |
| mean age  ≥ 50 years^b^ | All-CA | 26,050 (10) | 0.002 (0.439) | 0.002 (0.632) | 36.7(0.115) |  |
|  | All-CA^d^ | 17635 (8) | 0.007 (0.083) | 0.005 (0.351) | 34.9 (0.149) |  |
|  | GP | 19,953 (7) | 0.004 (0.301) | 0.003 (0.637) | 53.9 (0.043) |  |
|  | GP^g^ | 13221 (6) | 0.011 (0.037) | 0.006 (0.501) | 45.7 (0.101) |  |
| mean age  < 50 years^b^ | All-CA | 30434 (11) | 0.002 (0.440) | 0.002 (0.551) | 25.8 (0.198) | 0.954 [0.989] |
|  | All-CA^d^ | 16322 (7) | 0.001 (0.851) | 0.001 (0.851) | 0.0 (0.519) | 0.266 [0.513] |
|  | GP | 28,891 (9) | 0.003 (0.248) | 0.004 (0.270) | 14.7 (0.311) | 0.914 [0.970] |
|  | GP^g^ | 15825 (6) | 0.002 (0.639) | 0.002 (0.639) | 0.0 (0.771) | 0.176 [0.683] |
| BMI after/during year 2000^c^ | All-CA | 20,569 (9) | 0.010 (0.007) | 0.007 (0.175) | 39.2 (0.107) |  |
|  | All-CA^d^ | 14,917 (7) | 0.010 (0.037) | 0.006 (0.328) | 36.3 (0.151) |  |
|  | GP | 17,217 (7) | 0.013 (0.002) | 0.010 (0.121) | 41.2 (0.116) |  |
|  | GP^g^ | 13,221 (6) | 0.011 (0.037) | 0.006 (0.501) | 45.7 (0.101) |  |
| BMI  before year 2000^c^ | All-CA | 35,888 (12) | -0.001 (0.623) | -0.001 (0.623) | 0.0 (0.706) | 0.011 [0.150] |
|  | All-CA^d^ | 19,040 (8) | 0.000 (0.917) | 0.000 (0.917) | 0.0 (0.631) | 0.111 [0.416] |
|  | GP | 31,627 (9) | -0.000 (0.890) | -0.000 (0.890) | 0.0 (0.766) | 0.007 [0.138] |
|  | GP^g^ | 15,825 (6) | 0.002 (0.639) | 0.002 (0.639) | 0.0 (0.771) | 0.176 [0.683] |

^a^ Studies with percentage of subjects with BMI ≥ 30 ≥ 18% or < 18%. ^b^ Studies with mean age ≥ 50 years or < 50 years. ^c^ Studies with BMI assessment after/during year 2000 or before year 2000 ^d^ Excluding studies published before the response letter by Herbert et al., December 2006, in which the hypothesis of potential heterogeneity due to study design and a first call for a meta-analysis were stated (i.e. excluding American_Polish, NHS, KORA-S4, Essen_trios, EPIC_Norfolk, MRC_Ely, DESIR, SHIP, OB_adult). ^e^ Testing for difference of fixed effect [random effect] beta-estimate. All-CA = Caucasian adult studies, GP = General population studies.
