# Supplementary material for: Meta-Analysis of the INSIG2 Association with Obesity Including 74,345 Individuals: Does Heterogeneity of Estimates Relate to Study Design?
Source: PLoS Genet. 2009 Oct 23;5(10):e1000694. doi: 10.1371/journal.pgen.1000694 (PMC2757909; doi:10.1371/journal.pgen.1000694)
Supplement: Table S7 — Sensitivity analyses for the association of the INSIG2 SNP with BMI regarding sex, age, published status, or self-reported or measured BMI. Stated values are beta-estimates (p-values) based on fixed and random effects models, I2 (p-value of Q test) for each group, and p-values testing for difference between the beta-estimates of the two groups. (Not for HP, All-NC, or All-CH due to low numbers of studies.) (0.07 MB DOC) [file pgen.1000694.s008.doc]

**Table S7: Sensitivity analyses for the association of the *INSIG2* SNP with BMI regarding sex, age, published status, or self-reported or measured BMI.** Stated values are beta-estimates (p-values) based on fixed and random effects models, I² (p-value of Q test) for each group, and p-values testing for difference between the beta-estimates of the two groups. (not for HP, All-NC, or All-CH due to low numbers of studies)

| Group |  | # subjects  (# studies) | beta (p-value)  fixed effect | beta (p-value)  random effect | I² (p-value) | Testing for difference  p-value^e^ |
| --- | --- | --- | --- | --- | --- | --- |
| Men^a^ | All-CA | 27,826 (20) | 0.004 (0.132) | 0.004 (0.132) | 0.0 (0.583) |  |
|  | GP | 22,888 (16) | 0.004 (0.151) | 0.004 (0.160) | 8.7 (0.354) |  |
| Women^a^ | All-CA | 28,658 (20) | -0.000 (0.942) | -0.002 (0.206) | 47.2 (0.011) | 0.320 [0.267] |
|  | GP | 25,956 (16) | 0.003 (0.463) | 0.003 (0.360) | 24.4 (0.177) | 0.722 [0.723] |
| Age ≥ 50 yrs^b^ | All-CA | 30,215 (20) | 0.003 (0.340) | 0.003 (0.491) | 42.3 (0.024) |  |
|  | GP | 24,459 (15) | 0.005 (0.115) | 0.006 (0.182) | 40.8 (0.051) |  |
| Age < 50 yrs^b^ | All-CA | 26,267 (18) | 0.002 (0.634) | 0.002 (0.634) | 0.0 (0.734) | 0.783 [0.798] |
|  | GP | 24,385 (14) | 0.002 (0.519) | 0.002 (0.519) | 0.0 (0.541) | 0.529 [0.477] |
| Published^c^ | All-CA | 36,626 (12) | 0.002 (0.342) | 0.003 (0.338) | 30.0 (0.152) |  |
|  | GP | 31,179 (9) | 0.004 (0.160) | 0.006 (0.151) | 39.7 (0.103) |  |
| Unpublished^c^ | All-CA | 19,858 (9) | 0.002 (0.582) | -0.000 (0.952) | 33.1 (0.153) | 0.910 [0.546] |
|  | GP | 17,665 (7) | 0.003 (0.484) | 0.001 (0.895) | 33.9 (0.169) | 0.786 [0.433] |
| Self-reported BMI^d^ | All-CA | 3,001 (3) | -0.018 (0.057) | -0.018 (0.057) | 0.0 (0.981) |  |
|  | GP | 1955 (2) | -0.018 (0.097) | -0.018 (0.097) | 0.0 (0.876) |  |
| Measured BMI^d^ | All-CA | 53,483 (18) | 0.003 (0.118) | 0.004 (0.166) | 25.3 (0.157) | 0.027 [0.027] |
|  | GP | 46,889 (14) | 0.005 (0.054) | 0.005 (0.077) | 29.0 (0.146) | 0.043 [0.040] |

^a^ Combined results of study-specific analyses stratified for men or women. ^b^ Combined results of study-specific analyses stratified for subjects ≥ 50 years or < 50 years of age. ^c^ Combined results for the published or the unpublished studies. ^d^ Combined results for the studies with self-reported BMI or with measured BMI. ^e^ Testing for difference of fixed effect [random effects] beta estimates. All-CA = Caucasian adult studies, GP = General population-based studies.
