# Supplementary material for: Meta-Analysis of the INSIG2 Association with Obesity Including 74,345 Individuals: Does Heterogeneity of Estimates Relate to Study Design?
Source: PLoS Genet. 2009 Oct 23;5(10):e1000694. doi: 10.1371/journal.pgen.1000694 (PMC2757909; doi:10.1371/journal.pgen.1000694)
Supplement: Text S1 — References of included published studies; data form sent to each study partner; predefined analysis plan; amendment to analysis plan; and classification of studies due by study type. (0.18 MB DOC) [file pgen.1000694.s009.doc]

**Text S1**

**Contents**

1. References of included published studies page 2
2. Data form sent to each study partner page 4
3. Pre-defined Analysis Plan page 11
4. Amendment to Analysis Plan page 13
5. Classification of studies due to study type page 14

**A. References of included studies**

**B. Data form as sent out to study partners**

Dear colleagues,

Obesity is an increasing phenomenon throughout the world and a major risk factor for several chronic diseases such as type 2 diabetes mellitus and cardiovascular disease. The knowledge about the genetic distribution is just at a start. The *INSIG2* gene was discovered via a genome-wide SNP scan. Several replications confirm the finding, some do not. To clarify this association, we conduct a meta-analysis including all published and unpublished studies available, which are based on ***healthy populations, on the general population, or on studies specifically conducted for obesity***. We hope that you will join us in this ***INSIG2 rs7566605 meta-analysis for obesity****.*

In the primary report, the *INSIG2* rs7566605 showed association with obesity and body-mass-index (BMI) in a recessive model (*Herbert et al., Science, 2006*). The obesity-predisposing C/C genotype was present in about 10% of the individuals. The odds ratio (OR) for obesity (BMI>=30 kg/m²) was 1.22 for the C/C genotype compared to the G/G and C/G genotypes. This association was confirmed in 4 out of 5 replication samples. A further publication included 4 studies showing and 3 studies not showing the association (*Lyon et al., Plos Genetics 2007*). As the non-replication of initial findings is a well-acknowledged problem in genetic epidemiology due to false positives, heterogeneity between studies, or quality issues, we seek to provide a clear picture by conducting a meta-analysis of all available data.

This meta-analysis gives the opportunity for co-authorship when study inclusion criteria are met and the needed information is made available via e-mail ([heid@gsf.de](mailto:heid@gsf.de)) to the meta-analysis study center, which are the Institute of Epidemiology, GSF-National Research Center, Germany, and the Department of Biostatistics, Harvard Medical School, U.S.A. ***A check list of eligibility of your study and a list of the needed data information are provided below. You may also send your data, if you prefer*** (subject-ID, age [yrs], sex [1=men, 2=women], BMI [kg/m²], obesity [0=no, 1=yes)]**.** We will keep your data confidential until the meta-analysis is finalized. We will share the data with the other meta-analysis partners when the first publication manuscript is drafted.

If you know about other researchers who might be able to contribute, please let us know or give them our e-mail address to contact us.

We are looking forward to sharing the results with you. Thank you for your help!

With kind regards,

| Iris M. Heid, PhD  [heid@gsf.de](mailto:heid@gsf.de) +49 (0) 89 3187 3084 or +49 (0) 941 7994834  Cornelia Huth, MSE [huth@gsf.de](mailto:huth@gsf.de) +49 (0) 89 3187 4558 or +49 (0) 8252 881789  H.-Erich Wichmann, MD, PhD Professor [wichmann@gsf.de](mailto:wichmann@gsf.de)  GSF-National Research Center, Institute of Epidemiology, 85764 Neuherberg, Germany | Christoph Lange, PhD Professor [clange@hsph.harvard.edu](mailto:clange@hsph.harvard.edu)  Nan Laird, PhD Professor [laird@hsph.harvard.edu](mailto:laird@hsph.harvard.edu)  Department of Biostatistics,  Harvard School of Public Health,  Boston, MA 02115, USA |
| --- | --- |

***To-fill-in form on summary information for INSIG2 rs7566605 meta-analysis for obesity***

Please send to Iris M. Heid, [heid@gsf.de](mailto:heid@gsf.de)

**1.** Check of eligibility

| Is your study based on a ***healthy population, on a general population, or was it specifically conducted for obesity***?  If YES, your study is eligible.  If your study was conducted e.g. as a type 2 diabetes study, your study is NOT eligible. |  |
| --- | --- |
| Does your study include ***more than 200 subjects*** with data on age, sex, BMI, and rs7566605? If it is a case-control study, does it include more than 100 cases and more than 100 controls? If it is a family study, does it include more than 200 subjects altogether?  If YES, your study is eligible. |  |
| Does your study have ***ethical approval***?  If YES, your study is eligible. |  |

***If your study is not eligible for this meta-analysis, please note that you do not have to fill in this form any further. If you are unsure, whether your study is eligible, please feel free to e-mail to*** [***heid@gsf.de***](mailto:Heid@gsf.de) ***for further information.***

2. Study recruitment

| Name of study |  |
| --- | --- |
| Short name of study (maximum of 20 letters) |  |
| One author for this meta-analysis: name, e-mail, and affiliation |  |
| A second author for your study: name, e-mail and affiliation* |  |
| Is your study published/submitted for association of *INSIG2* rs7566605 with obesity? (Indicate reference)  If yes, are study participants here the same as in the published reference? |  |

* If less than 25 studies will be collected for this meta-analysis, two authors per study can be co-author of this meta-analysis. Otherwise, only one author per study can be included to not exceed 50 authors. In any case, further persons can be stated in the acknowledgements.

**3. Study design**

- If you use the baseline survey of a cohort study, please indicate it as a cross-sectional study (BMI can be analyzed as a quantitative trait).
- If you use a cross-sectional study to yield a case-control sample and you have genotyped all subjects, please indicate it as a cross-sectional study (BMI can be analyzed as a quantitative trait).
- If you use a cross-sectional study to yield a case-control sample and you have only genotyped a part of your cross-sectional study skipping a part of the BMI scale (e.g. only genotyped subjects with BMI ≥ 35 and BMI < 25 kg/m²), please indicate it as case-control study (BMI will only be analyzed as a dichotomous trait).
- For case-control studies with cases and controls derived other than from one cross-section of a population, BMI will not be analyzed quantitatively.

| Your study is a *cross-sectional study* / *case-control study* / *family study* / *other* (please state)? |  |
| --- | --- |
| For *cross-sectional*: Please indicate: population*, recruitment, sample size. |  |
| For *case-control*: Please indicate for cases: population*, recruitment, number of cases, BMI cutpoint, and for controls: population*, recruitment, number of controls, BMI cutpoint.  Please indicate matching procedure, if applicable. |  |
| For *family study*: Please indicate: population* of index probands, recruitment, number of index probands, structure of pedigrees**, total number of subjects in study. |  |
| For other designs: Please describe. |  |
| What is the ethnicity of your participants? |  |
| When did you assess the BMI or obesity status of your study participants (calendar year)? |  |
| Does your study include children (age < 18 yrs)? Please state their number. |  |

* Indicate your population as general population / healthy population / health professionals / working population / children / obese subjects (indicate BMI cut-off point)

** Structure of pedigrees may be: sibs / parents only / parents&sibs / full pedigrees of living relatives.

**4. Phenotyping and genotyping quality information**

| BMI was measured* / self-reported** |  |
| --- | --- |
| Method of genotyping |  |
| Call rate (e.g. 95.55%) |  |

* i.e. calculated from measured height in cm and measured weight in kg

** either weight or height or both were self-reported by the study participants

**5. Genotype frequencies**

Please note that all numbers relate to the analyzed sample, which consists of the subjects with non-missing data on BMI, age, sex, and rs7566605 genotype.

|  |  | N  C/C genotype | N  C/G genotype | N  G/G genotype |
| --- | --- | --- | --- | --- |
| Example | N Obese* | 80 | 420 | 350 |
|  | N Non-obese* | 350 | 1800 | 1300 |
| Your study | N Obese* |  |  |  |
|  | N Non-obese* |  |  |  |

* “Obese” indicates BMI ≥ 30.0 kg/m², and non-obese BMI < 30.0 kg/m². If this is not possible, see table below.

If your study covers the full range of BMI, please fill in all the cells of the two tables below. If your case-control study has a BMI cut-off point for cases that is higher than 30 kg/m² and the middle range is not available, then please indicate this in the study design section 3 and fill in the most appropriate row in the table below.

|  |  | N  C/C genotype | N  C/G genotype | N  G/G genotype |
| --- | --- | --- | --- | --- |
| Your study | N with BMI ≥ 32.5 kg/m² |  |  |  |
|  | N with BMI ≥ 35.0 kg/m² |  |  |  |
|  | N with BMI ≥ 37.5 kg/m² |  |  |  |
|  | N with BMI ≥ 40.0 kg/m² |  |  |  |

|  |  | N  C/C genotype | N  C/G genotype | N  G/G genotype |
| --- | --- | --- | --- | --- |
| Your study | N with BMI < 25 kg/m² |  |  |  |
|  | N with BMI < 20 kg/m² |  |  |  |

**6. Covariate characteristics**

Please note that all numbers relate to the analyzed sample, which consists of the subjects with non-missing data on BMI, age, sex, and rs7566605 genotype.

For cross-sectional study:

|  | N females | N  males | N age<50yrs | N  age≥50yrs | Age [yrs]  Mean±SD  (min-max) | BMI [kg/m²]  Mean±SD  (min-max) |
| --- | --- | --- | --- | --- | --- | --- |
| Example | 2000 | 2000 | 2000 | 2000 | 50.45±10.36 (25.00-75.00) | 35.25±4.60 (17.46-45.50) |
| Yourstudy |  |  |  |  |  |  |

For case-control study:

|  |  | N females | N  males | N  age <50yrs | N  age >=50yrs | Age [yrs]  Mean±SD  (min-max) | BMI [kg/m²]  Mean±SD (min-max) |
| --- | --- | --- | --- | --- | --- | --- | --- |
| Example | cases | 500 | 500 | 500 | 500 | 50.45±10.36 (25.00-75.00) | 35.25±4.60 (30.00-45.50) |
|  | controls | 1500 | 1500 | 1500 | 1500 | 50.35±10.26 (25.00-75.00) | 24.45±4.45 (17.60-42.90) |
| Yourstudy | cases |  |  |  |  |  |  |
|  | controls |  |  |  |  |  |  |

For family study:

|  |  | N females | N  males | N  age<50yr | N  age >=50yrs | Age [yrs]  mean±SD (min-max) | BMI [kg/m²]  mean±SD  (min-max) |
| --- | --- | --- | --- | --- | --- | --- | --- |
| Yourstudy | index |  |  |  |  |  |  |
|  | others |  |  |  |  |  |  |

**7. Association analyses**

Please apply a recessive model and test the C/C genotype (homozygous of the minor allele) versus the C/G or GG genotype.

Dichotomous trait “obesity” (for cross-sectional, for case-control, and for family studies): Obese should be defined, if possible, as BMI ≥ 30 kg/m² (OBESITY=1) and non-obese as BMI < 30 kg/m² (OBESITY=0). Please compute the beta estimate (natural logarithm of odds ratio) and the corresponding standard error (SE) via logistic regression adjusted for age and sex. For family studies, please apply conditional logistic regression.

Quantitative trait BMI (for cross-sectional studies, for family studies): Please compute beta estimate and the corresponding standard error (SE) via linear regression adjusted for age and sex with ***BMI on the ln scale***. For family studies, please apply regression conditioning on family.

**7.1. Results of main association analysis**

Dichotomous trait “obesity” (for cross-sectional, case-control, or family studies)

|  | Beta estimate (lnOR)* | SE |
| --- | --- | --- |
| Example | 0.1655 | 0.0830 |
| Yourstudy |  |  |

If you have a case-control study with the cut-off point for cases being higher than 30 kg/m² or for controls being lower than 30 kg/m², then please indicate this in the study design section and insert the lnOR and the SE here nevertheless.

* Via logistic regression adjusted for age and sex with recessive genetic model

Quantitative trait “BMI” (for cross-sectional or family studies)

|  | Beta estimate* | SE |
| --- | --- | --- |
| Example | 0.0234 | 0.0100 |
| Yourstudy |  |  |

Please use BMI on the ln scale.

* Via linear regression adjusted for age and sex with recessive genetic model

**7.2. Results for various degrees of obesity**

If your study covers the full range of BMI, then please fill in all the rows and columns in the table below. If your study does NOT cover the middle range of BMI (e.g. case-control studies with a cut-off point for obesity higher than 30 kg/m²), please fill in the rows and columns that are appropriate for your study.

|  | Degree of obesity | Ln(OR)  Compare to BMI <30 | SE  Compare to BMI <30 | Ln(OR)  Compare to BMI <25 | SE  Compare to BMI <25 | Ln(OR)  Compare to BMI <20 | SE  Compare to BMI <20 |
| --- | --- | --- | --- | --- | --- | --- | --- |
| Yourstudy | BMI ≥ 30.0 |  |  |  |  |  |  |
|  | BMI ≥ 32.5 |  |  |  |  |  |  |
|  | BMI ≥ 35.0 |  |  |  |  |  |  |
|  | BMI ≥ 37.5 |  |  |  |  |  |  |
|  | BMI ≥ 40.0 |  |  |  |  |  |  |

* Via logistic regression adjusted for age and sex with recessive genetic model

**7.3. Results of subgroup analyses**

Dichotomous trait obesity (as in 7.1.):

|  | Subgroup | Adjusted for | Ln(OR) | SE |
| --- | --- | --- | --- | --- |
| Yourstudy | men | age |  |  |
|  | women | age |  |  |
|  | age < 50 yrs | sex |  |  |
|  | age ≥ 50 yrs | sex |  |  |

Quantitative trait BMI (as in 7.1):

|  | Subgroup | Adjusted for | Linear association estimate | SE |
| --- | --- | --- | --- | --- |
| Yourstudy | men | age |  |  |
|  | women | age |  |  |
|  | age < 50 yrs | sex |  |  |
|  | age ≥ 50 yrs | sex |  |  |

THANK YOU FOR YOUR TIME AND EFFORT!

**C. Pre-defined analysis plan**

***INSIG2* Meta-Analysis**

**Iris M. Heid, Cornelia Huth,
Nan Laird, Christoph Lange, H.-E. Wichmann**

**First version sent out to partners 07/2007**

**Updates 02/2008 (Heid)**

| Iris M. Heid, PhD  [heid@gsf.de](mailto:heid@gsf.de) +49 (0) 89 3187 3084 or +49 (0) 941 7994834  Cornelia Huth, MSE [huth@gsf.de](mailto:huth@gsf.de) +49 (0) 89 3187 4558 or +49 (0) 8252 881789  H.-Erich Wichmann, MD, PhD Professor [wichmann@gsf.de](mailto:wichmann@gsf.de)  GSF-National Research Center, Institute of Epidemiology, 85764 Neuherberg, Germany | Christoph Lange, PhD Professor [clange@hsph.harvard.edu](mailto:clange@hsph.harvard.edu)  Nan Laird, PhD Professor [laird@hsph.harvard.edu](mailto:laird@hsph.harvard.edu)  Department of Biostatistics,  Harvard School of Public Health,  Boston, MA 02115, USA |
| --- | --- |

**(1) Aim of this project:** To provide conclusive evidence about an association of the *INSIG2* rs7566605 and BMI or obesity and to help understand the heterogeneity between study-specific association estimates.

Regarding heterogeneity we will investigate a potential effect of:

- age (outcomes BMI and obesity)
- sex (outcomes BMI and obesity)
- study populations: general population, healthy population or obesity study (outcome BMI and obesity)
- proportion of obese subjects in population (outcome BMI)
- cut-off point for definition of obesity as the association might only be present when comparing more extreme cases with controls (outcome obesity)
- year of BMI phenotyping as there might be an effect modification by an environmental factor which has changed over time (outcomes BMI and obesity)

**(2) Study inclusion criteria for meta-analysis:** All human epidemiological studies with the *INSIG2* rs7566605 genotyped and BMI, sex and age available including >200 subjects (for Ccs >100 cases and >100 controls) from the general or healthy population or studies explicitely selected for obesity, except for the initial FHS screen sample. A sample is considered to be from a “healthy population” if the sample stems from a working population or if subjects with rare diseases are excluded (e.g. a sample excluding MI subjects).

The meta-analysis thus ***includes*** three different types of study populations:

(a) general population (GP) studies for both quantitative and dichotomous analysis including

(i) cross-sectional, (ii) family study designs

(b) healthy population (HP) studies for both quantitative and dichotomous analysis including

(i) cross-sectional, (ii) family study designs

(c) obesity-ascertained studies (O) for dichotomous analysis:

(i) case control, (ii) family study designs

The meta-analysis thus ***excludes***:

(a) the initial FHS screen

(b) studies ascertained for disease, as metabolic mechanisms are considered to be very different in patient populations and it is difficult to allow for one disease, but rule out another as it would not be clear where to draw the line

(c) studies using only non-diabetic subjects, as this would imply that a great proportion of the upper BMI percentiles is missing compared to a GP sample.

**(3) Study recruitment**

To avoid bias, we have separated the tasks during study recruiting phase. One investigator (Heid) is responsible for study recruitment without looking at the incoming data; another investigator (Huth) is responsible for data checking and database entry.

A systematic Pubmed literature search was conducted. Investigators of all published studies (as of 12/2007) were contacted. Several consortial groups were contacted (GIANT, *IL6* joint analysis, German KORA 500K Project) for recruitment of unpublished studies.

**(4) Planned analyses**

(A) General population (GP) analysis: In order to derive whether there is an association in general, we will provide association estimates including all ***general population studies of adult Caucasians*** for (i) quantitative BMI, (ii) dichotomous obesity, (iii) varying cutpoints for obesity. This approach was chosen due to the large number of subjects from GP studies (estimated n=50,000, 18 studies), due to the few studies with children (3 studies) or other ethnicities (4 studies), and due to the prior believe of general population-based studies being the most homogeneous and the least threatened by bias. (The studies on healthy populations and obesity studies are very important on the other side to complement the picture and particularly the obesity studies may be best suited to depict an effect in the extremes)

Test on 5% level of statistical significance.

(B) Full sample analysis: In order to investigate the previously observed heterogeneity between published association estimates, the analysis is extended to include ***adult Caucasians*** from

- ***healthy populations***: control groups for MI, sample excluding CVD, healthy exercise study, healthy working populations (NHS, SAPHIR)
- ***obesity studies***: family and case control studies for obesity including extreme obesity and Utah gastric bypass study

Check whether there is heterogeneity due to study population (GP, HP, O; 10% level).

Test on 1.65% level for statistical significance in either of the two additional groups.

(C) Check in GP or in full sample (depending on (B)), whether there is heterogeneity due to the following ***subgroups of studies***

(ii) “old” versus “young” adult study population (i.e. studies with median age above average versus below average).

(iii) “thick” versus “lean” study population (i.e. studies with median BMI above average versus below average)

(iv) “recent” studies versus “more ancient” studies (i.e. studies with BMI phenotyping after or before the year 2000)

Heterogeneity is tested to 10% level; statistical significance in either of the groups to 0.6% (nine groups so far).

(D) ***Sensitivity analyses:***

(i) ***Subgroup of subjects*** (in GP or full sample depending on (B)): “old” versus “young” adults (i.e. separate subjects above or below 50 years of age); women versus men

(ii) Analyze whether the effect pertains/appears in ***children and other ethnicities***.

(iii) including studies with HWE violation (if applicable)

(iv) excluding studies with self-reported BMI

**(7) Conclusions to be drawn**

***If we detect a statistically significant (5% level) association in the GP analysis in the direction of the initial FHS screen (i.e. increased risk of obesity for minor allele) in step A:***

We would conclude that there is an association and that the initial report is confirmed. If subsequent analysis (B, C and D step) would show that heterogeneity is due to any of the tested characteristics, we would conclude that some of the heterogeneity is explained.

***If we do not find a statistically significant association in the direction of the initial FHS screen in step A:***

If we subsequently find heterogeneity in step B due to the predefined characteristics and significant association in one of the subgroups (to the multiple testing corrected significance level), we will conclude that there is effect, but masked by heterogeneity.

If we subsequently do not find heterogeneity and no significant association in the a priori defined subgroups in step B, we will conclude that we could not detect an apparent effect.

**D. Amendment to Analysis Plan**

In the review process of this work, we decided **to start with a combined analysis of all available studies on Caucasian adults followed by a stratified analysis of these by study design** (GP = general population, HP = healthy population, OB = obesity study), **as well as a combined analysis of available studies on Non-Caucasian adults and the combined analysis of the children studies**. The reason for this change in the analysis plan was to give the most objective picture possible of this *INSIG2* SNP association based on our data.

We also gave credit to the fact that the studies published before the *Science* letter by Herbert et al., December 2006, which was a response to various publications with inconsistent results, motivated four of our five hypotheses for a potential source of heterogeneity of the *INSIG2* SNP association. In fact, in this letter, a first call for this meta-analysis was stated. Therefore, we provide combined meta-analysis results also excluding the studies published before this letter (i.e. excluding American_Polish, NHS, KORA_S4, Essen_trios, EPIC_Norfolk, MRC_Ely, DESIR, SHIP, OB_adult).

**E. Classification of studies by study type**

The classification of the study as ***general population-based (GP) study***, ***healthy population (HP) study***, or ***obesity study (OB)*** was performed a-priori with as much control for classification bias as possible by blinding the scientist making the classification decision (I.M.H) for study name and association results, while the other scientist (C.H.) presented information on the study design. After the initial classification and the end of the blinding of I.M.H., we did not change this classification. A potential mis-classification cannot be completely ruled out, which could dilute the association. However, an informative mis-classification (i.e. informative about the direction of the association) and thus a bias away from the null hypothesis was thereby made highly unlikely.

The precise definitions of the three study types were as follows:

***GP studies*** are studies based on a general population of a certain age range without any exclusions related to a better health (otherwise they would have been classified HP) or exclusions related to disease or disease-related conditions (otherwise they would not have been included in this meta-analysis at all). The KIEL_AGING study might be considered a very healthy population as the subjects were all about 100 years old and thus might be viewed to survive better than the general population, but it was not considered a HP study here based on our definition as GP based on ‘a certain age range’. (The association results would not be affected qualitatively by a re-classification of this study as HP).

***HP studies*** are studies ascertaining subjects for reasons related to a better health status: E.g. studies with purely subjects in the work force such as nurses (NHS) or the general work force in Salzburg by recruiting through company physicians (SAPHIR) were classified as HP. A study was also classified as HP if it was a general population-based study but excluded subjects previously diagnosed with type 2 diabetes (MRC-ELY), if it excluded subjects with previous myocardial infarction, cerebrovascular disease, or life-threatening malignancies (NPHSSI), or excluded subjects with several disease-related conditions to particularly recruit ‘healthy subjects’ (Heritage_White). Some studies classified as HP by our strict definition might be considered GP in a different context (i.e. MRC-ELY).

***OB studies*** are studies that were specifically designed to investigate obesity. Only one was a family-based study recruiting obese index subjects and their families (OB_adult). The others were obesity case-control studies recruiting ‘cases’ from adiposity ambulant services (Essen_obese, Obenutic, Swiss_obese), among subjects seeking consultancy for obesity surgery (UTAH), or from the extremes of general-population studies (American_Polish).
